# Intravitreal injection of fibrillin 2 (Fbn2) recombinant protein for therapy of retinopathy in a retina-specific Fbn2 knock-down mouse model

**DOI:** 10.1038/s41598-023-33886-6

**Published:** 2023-04-26

**Authors:** Rui Xue Zhang, Ying Wen, Da Dong Guo, Fu Ru Xu, Gui Min Wang, Xing Rong Wang, Yong Wei Shi, Jie Ding, Qian Jiang, Wen Jun Jiang, Jost B. Jonas, Hong Sheng Bi

**Affiliations:** 1 Shandong University of Traditional Chinese Medicine, Jinan, China; 2 Affiliated Eye Hospital of Shandong University of Traditional Chinese Medicine, Jinan, China; 3 Shandong Provincial Key Laboratory of Integrated Traditional Chinese and Western Medicine for Prevention and Therapy of Ocular Diseases, Key Laboratory of Integrated Traditional Chinese and Western Medicine for Prevention and Therapy of Ocular Diseases in Universities of Shandong, Shandong Academy of Eye Disease Prevention and Therapy, Jinan, China; 4 Department of Ophthalmology, Medical Faculty Mannheim of the Ruprecht-Karls-University Heidelberg, Mannheim, Germany

**Keywords:** Drug discovery, Genetics, Molecular biology, Physiology, Medical research, Molecular medicine

## Abstract

Mutations in the extracellular matrix gene Fibrillin-2 (FBN2) are related to genetic macular degenerative disorders including age-related macular degeneration (AMD) and early-onset macular degeneration (EOMD). It was reported that the retinal protein expression of FBN2 was reduced in patients with AMD and EOMD. The effect of exogenously supplied fbn2 recombinant protein on fbn2-deficiency-related retinopathy was not known. Here we investigated the efficacy and molecular mechanism of intravitreally applied fibrin-2 recombinant protein in mice with fbn2-deficient retinopathy. The experimental study included groups (all n = 9) of adult C57BL/6J male mice which underwent no intervention, intravitreal injection of adeno-associated virus (AAV) empty vector or intravitreal injection of AAV-sh-fbn2 (adeno-associated virus for expressing short hairpin RNA for fibrillin-2) followed by three intravitreal injections of fbn2 recombinant protein, given in intervals of 8 days in doses of 0.30 μg, 0.75 μg, 1.50 μg, and 3.00 μg, respectively. Eyes with intravitreally applied AAV-sh-fbn2 as compared to eyes with injection of AAV-empty vector or developed an exudative retinopathy with involvement of the deep retinal layers, reduction in axial length and reduction in ERG amplitudes. After additional and repeated application of fbn2 recombinant protein, the retinopathy improved with an increase in retinal thickness and ERG amplitude, the mRNA and protein expression of transforming growth factor-beta (TGF-β1) and TGF-β binding protein (LTBP-1) increased, and axial length elongated, with the difference most marked for the dose of 0.75 μg of fbn2 recombinant protein. The observations suggest that intravitreally applied fbn2 recombinant protein reversed the retinopathy caused by an fbn2 knockdown.

## Introduction

Missense mutations in the extracellular matrix gene Fibrillin-2 *fbn2* have been reported to be associated with age-related macular degeneration (AMD) and early-onset macular degeneration (EOMD)^1^. Fibrillin is a glycoprotein, which is essential for the formation of elastic fibers in connective tissue^2^. Secreted into the extracellular matrix by fibroblasts, it gets incorporated into the insoluble microfibrils, forming a scaffold for the deposition of elastin. Three forms of fibrillin have been described so far. Fibrillin-1 is included in microfibrils creating a sheath around amorphous elastin, and mutations in its FBN1 gene cause Marfan syndrome^3^. Fibrillin-2 has been discussed to be involved in early elastogenesis (i.e., the formation and development of elastic fibers), in which microfibers consisting of FBN2 and FBN1 form a complex with tropoelastin and Fibulin4/5 and influence the elasticity of retinal pigment epithelium (RPE) layer, choroid and Bruch's membrane (BM)^4^. Mutations in the gene of fibrillin-2 have been linked to EOMD^1^. The reduction or loss of FBN2 compromised the barrier properties of BM, potentially associated with the pathogenesis of AMD^1^. Fibrillin-3 has been reported to be located mostly in the brain^5^.

Adeno-associated virus (AAV) is a widely used, non-pathogenic vector with a long-term transgene expression effect and which confers a relatively low immunogenic response in privileged areas such as the eye and brain^6^. In the case of a genetic disease with the loss, or production of a misshaped, protein, therapeutic procedures have been aimed to substitute the missing or misshaped protein by a recombinant protein. In the case of the *fbn2* deficiency retinopathy, it would thus be reasonable to substitute the lacking fibrillin-2 protein by an intravitreally applied recombinant protein. We therefore conducted this study to explore the effect and molecular mechanism of intravitreally injected fbn2 recombinant protein for the treatment of fbn2-deficiency retinopathy. We developed an AAV-knockdown *fbn2-*deficiency retinopathy mouse model by intravitreally applied AAV-sh-fbn2 (adeno-associated virus for expressing short hairpin RNA for fibrillin-2), and then injected the *fbn2* recombinant protein in various doses. We assessed changes in the appearance of the ocular fundus, performed biometric and histological measurements, and carried out electroretinographical and immunohistochemical examinations. The findings may provide new insights for the understanding of molecular mechanism of *fbn2*-deficiency retinopathy in particular and for the etiology of macular degeneration in general, and they may be helpful for the development of new drugs for the treatment of inherited retinal degenerations.

## Methods

### Animals

The experimental study included adult C57BL/6J male mice with an age of 8 weeks and a body weight of 22–23 g (supplied by Beijing Vital River Experimental Animal Technology Co. Ltd., Beijing, China.)^7^. The Ethics committee of the Eye Institute of the Shandong University of Traditional Chinese Medicine approved the study and confirmed that its design was in accordance with ARVO (Association for Vision and Eye Research) statement for the use of Ophthalmic and Visual Research. The report in the manuscript follows the recommendations of the Animal Research: Reporting of In Vivo Experiments (ARRIVE) guidelines. All animals were housed and bred in an animal room and exposed to a 12 h light/dark cycle with free access to food and water. The whole group of animals was divided into several groups:

- Group #1—Animals without intervention (n = 9 animals).
- Group #2—Animals with an intravitreal injection of AAV empty vector and without any further treatment (n = 9 animals).
- Group #3—Animals with an intravitreal injection of AAV-sh-fbn2 and without any further treatment (n = 9 animals).
- Group #4—Animals with an intravitreal injection of AAV-sh-fbn2, followed by an intravitreal injection of fbn2 recombinant protein in a dose of 0.30 μg (n = 9 animals).
- Group #5—Animals with an intravitreal injection of AAV-sh-fbn2, followed by an intravitreal injection of fbn2 recombinant protein in a dose of 0.75 μg (n = 9 animals).
- Group #6—Animals with an intravitreal injection of AAV-sh-fbn2, followed by an intravitreal injection of fbn2 recombinant protein in a dose of 1.50 μg (n = 9 animals).
- Group #7—Animals with an intravitreal injection of AAV-sh-fbn2, followed by an intravitreal injection of fbn2 recombinant protein in a dose of 3.00 μg (n = 9 animals).

**Figure 1:**
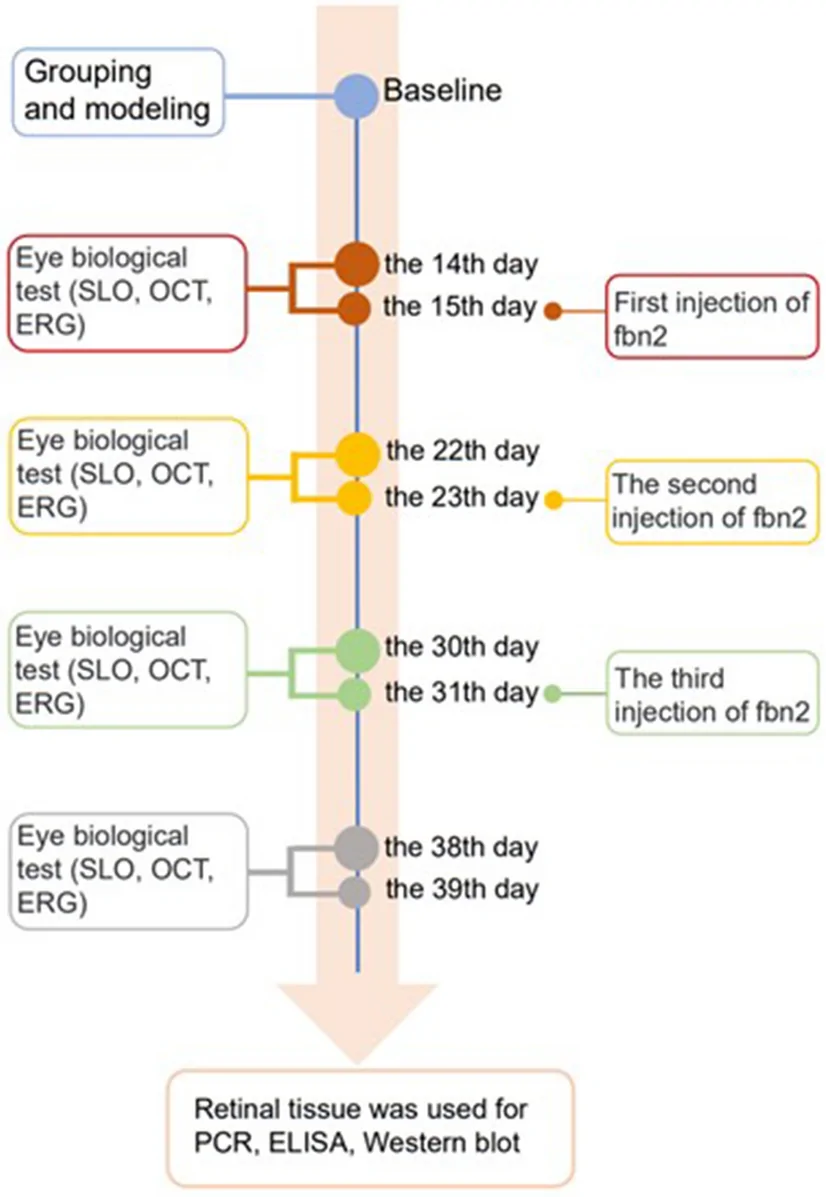
Experimental time node flow chart.

All injections were performed in the right eyes, while the left eyes remained untouched. At baseline of the study and at each follow-up examination (i.e., at 14 days after the AAV-sh-fbn2 injection, and at 7 days after the fbn2 recombinant protein injection), the animals underwent a series of examinations including confocal laser scanning laser ophthalmoscopy of the fundus to obtain wide-field images of the fundus (Scanning Laser Confocal Ophthalmoscope (SLO), Daytona device, Optima, Marlborough, MA, USA), optical coherence tomography (OCT) of the fundus (Spectralis^®^, Heidelberg Engineering, Heidelberg, Germany), retinal electrography (ERG) (Optoprobe Science Ltd., OPTO-III, Britain), and ocular biometry for measurement of axial length (Visante OCT, ZEISS, Oberkochen, Germany). Since the examination took two days, the total study duration was 39 days (Fig. 1). The examinations were performed in medical mydriasis (induced by topically applied tropicamide eye drops (Santen Pharmaceutical Co., Ltd. Shiga plant Japan)) and in general anesthesia (achieved by an intraperitoneal injection of 1% sodium pentobarbital solution (Shandong Xiya Regent Co., Ltd, Shan Dong, China) (50 mg/kg body weight)). During the OCT and SLO examinations, the mice were manually kept in front of the device in a position to ensure that the corneal vertex was aligned with the instrument, and that the instrument could automatically detect the light reflection signal of the corneal vertex. In addition, the fundus image shown on the device monitor served to properly hold to animal in front of the camera. It also helped to have a similar alignment at different time points of the study period. The SLO examinations were repeated three times for each examination. For the OCT examination, a 25-diopter lens was fitted on a 30° field of view objective lens, and a contact lens was placed on the cornea. A horizontal and vertical scan line was centered on the optic nerve head. The OCT cross-sectional images were analyzed with the HEYEX software. The retinal thickness was measured at a location of 3000 μm distant of the optic disc. The ERG examination consisted of a scotopic part with a stimulus intensity of 0.03 log cd-s/m^2^ for the assessment of the dark adaptive rod cell response, and with a stimulus intensity of 3 log cd-s/m^2^ with inter-stimulus intervals of 30 s for the assessment of the dark adaptive mixed cell response. After the scotopic ERG measurements, the mice were adapted under Ganzfeld light conditions of 30 cd-s/m^2^ for 10 min, and a photopic ERG was recorded. The amplitude of the ERG a-wave was defined as the distance from the baseline to the a-wave valley, and the amplitude of b-wave was measured as the distance between the troughs and peaks. All ERG measurements were performed by the same examiner (RXZ) at the same daytime.

### Intravitreal injections

To achieve a knock-down of the retinal *FBN2* gene, we intravitreally injected 1012 genome copies of the AAV vector “AAV9-shRNA-fbn2 (5′-GCACATACAATGTCGGCAAAG-3” with a viral titer of 1.14 × 1012 Vector Genomes per mL(VG /mL), or an AAV9-empty vector (AAV-EV) (Genomeditech, Shanghai, China). The injections were performed using Hamilton micro needles (Hamilton^®^ Microliter™ syringe, removable needle 701 ASRN, volume 5 μL, needle size 23 s–26 s ga (cone tip), needle L 43 mm; Sigma-Aldrich, St. Louis, MO, USA), after the periorbital region and the ocular surface had been disinfected by an iodine containing solution and after the periorbital region had been covered with a sterile drape. The injections were performed between the corneal limbus and the equator of the eyes and were directed towards the posterior pole of the eyes. Only the data of eyes with successful injections, i.e., without crystalline lens opacities after the injection and without leakage at the injection site were included into the statistical analysis. Before the injection and at the first day after the injection, antibiotic eye drops (ofloxacin eye drops, Santen Pharmaceutical Co., Ltd. Shiga, Japan) were applied three times per day.

Two weeks after the AAV-sh-fbn2 injection, the animals of groups #4 to #7 received intravitreal injections of the recombinant fbn2 protein in four concentrations (dose: 0.30 μg (concentration: 0.1 μg/μL; volume: 3 μL) (group #4); 0.75 μg (concentration: 0.25 μg/μL; volume: 3 μL) (group #5); 1.5 μg (concentration: 0.50 μg/μL; volume: 3 μL) (group #6); and 3 μg (concentration: 1 μg/μL; volume: 3μL) (group #7) (Catalog Number: IC8368; Accession # P31955; R&D Systems, Immuno Clone., Houston, USA), respectively^8^. The fbn2 protein preparation had a purity of > 97%. The source was Echerichia oli, the residues was Thir1550 ~ Cys1791, and the predicted molecular mass was 56 kDa. The protein diluent was Ringer's solution (Otsuka, China Otsuka Pharmaceutical Co., Ltd., Tianjin, China). According to the supplier's brochure, it neutralized the biological activity of *FBN2*, with the amino acid sequences of human *FBN2* and mice *FBN2* being similar. In a pre-study investigation, the therapeutic effect for intravitreal injections of fbn2 recombinant protein was highest for a series of 3 injections (Supplementary Table 1; Supplementary Fig. 1). The intravitreal fbn2 recombinant protein injections were repeated twice in intervals of 8 days. And protein intravitreal injection was performed 3 times^9^. The animals were sacrificed after the last injection, i.e., 39 days after the first injection of the AAV-sh-fbn2 (Fig. 1). The eyeballs were harvested and the retinas were gently excised and stored in at − 80 °C. To examine the expression of *FBN2*, transforming growth factor-beta 1 (TGF-β) and latent TGF-beta binding protein (LTBP-1), 12 retina tissues from each group were dissected for the molecular examination of RT-PCR and western blot (1:1), respectively.

### Reverse transcriptase polymerase chain reaction (RT-PCR)

Total RNA was extracted from tissues or cultured cells using Trizol reagent (Invitrogen, 15596026, CA, USA), and 1–2 μg of the RNA was used for reverse transcription examination using a reverse transcript kit (TaKaRa Bio Inc, TaKaRa, 6210A, Japan). Quantitative real-time PCR (qRT-PCR) analysis was performed using the SYBR Green I Master kit (Roche, 4707516001, Roche, Basel, Switzerland). Diluted cDNA was used in a 20μL real-time PCR reaction in duplicate for each gene. Cycle parameters were 95 °C for 5 min hot start and 45 cycles of 95 °C for 5 s, 55 °C for 10 s and 72 °C for 20 s. Blank controls with no cDNA templates were performed to rule out contamination. The specificity of the PCR product was confirmed by melting curve analysis. Primers for FBN2 were: forward seq., 5′-GTGTAACTGCCCGCCTGACTTCC-3′, reverse seq., 5′-ACCTACGCCGACCTCTGTGTTGC-3′. Primers for endogenous TGF-β1 were: forward seq., 5′-CTCCCGTGGCTTCTAGTGC-3′, reverse seq., 5′-GCCTTAGTTTGGACAGGATCTG-3′. Primers for endogenous LTBP-1 were: forward seq., 5′-TGGCCCAGAAACAGACCCTTACTT-3′, reverse seq., 5′-ACACAGCGGCCGTTTTCACAA-3′. Primers for endogenous Beta-actin were: forward seq., 5′-GTGAGCCTTCTTCCTGTTAG-3′, reverse seq., 5′-CATTCAGCTCCGCAAGACTT-3′. The expression levels of all genes were normalized to that of the house keeping gene beta actin. Relative gene expression levels were calculated by the formula 2−∆Ct, where △Ct (Critical threshold) = Ct of genes of interest − Ct of beta-actin. Fold changes of gene expression levels in the test groups relative to corresponding normal control groups were calculated by 2−∆Ct method as previously described.

### Enzyme linked immunosorbent assay (ELISA)

Retinas were homogenized in RIPA Lysis Buffer (Servicebio, Wuhan, China) containing 100 mmol phenylmethylsulphonyl fluoride under liquid nitrogen until the bulks were invisible. Further, samples were sonicated on ice for 20 min. After centrifugation at 6000 g for 10 min, the supernatants were collected and placed in a 96-well plate (NEST Biotechnology, Wuxi, China), and the protein level was quantified using the BCA concentration kit (Sparkjade, Shandong Cisco Czech Scientific Instrument Co., Ltd., Shandong, China). The contents of FBN2, TGF-β1, and LTBP-1 proteins were quantified using a commercially available assay kit from Shanghai Jianglai Biological Co., Ltd., (Shanghai, China). Briefly, the microtiter plate wells were coated with an antibody those cross-reacts with mouse FBN2, TGF-β1, and LTBP-1, respectively. The measurements were performed following by the manufacturer's instructions. Finally, the absorbance value was recorded using a microplate reader (Biotech Elx800, VT, USA) at a wavelength of 450 nm.

### Western blot

The expressions of FBN2, TGF and LTBP proteins in the retinas of mice in groups #1, #2, #3 and #5 (n = 9 eyes / group) were measured after the third intravitreal injection of fbn2 recombinant protein. The retina tissues were covered with RIPA lysis buffer supplemented with 1 mM of phenylmethylsulphonyl fluoride in accordance with the weight to volume in a ratio of 1:10 (mg:μl) and add liquid nitrogen for grinding, followed by the centrifugation at 8000 g for 5 min to collect the supernatants for the Western blot analysis. We used 12% sodium dodecyl sulfate polyacrylamide gel electrophoresis (SDS-PAGE) and polyvinylidene difluoride membrane for separating and transferring the samples. Primary antibodies against FBN2 (mouse# sc-393968,1:1000, Santa Cruz Biotechnology, USA), TGF (1:2000, Rabbit, ab215715, Abcam Co., Cambridge, MA, USA), LTBP-1 (Rabbit, 1:1000, ab78294, Abcam Co., Cambridge, MA, USA), and β-actin (dilution 1:10,000, Bioss antibodies, Beijing, China) were incubated overnight with the membranes at 4 °C. Finally, the membranes were incubated for 2 h at room temperature with 1:2000 IRDye 800CW goat anti-rabbit IgG (1:2000, zb2301) or 1:2000 IRDye 800CW goat anti-mouse IgG (1:2000, zb2305) antibodies. Finally, visualization was performed with DAB (Sigma) by a FUSION-FX7 imaging system (Vilber Lourmat, Marne-la-Vallée, France) and quantification was carried out by Fusion CAPT Software (Vilber Lourmat, France).

### Statistical analysis

Using a statistical software package (SPSS for Windows, version 27.0, SPSS, Chicago, IL), we determined the mean ± standard deviation of the main outcome parameters. For the comparison of the different groups, we first used an analysis of variance (ANOVA) to detect significant differences. In a second step, we applied student´s t-test for independent samples to further assess the significance of differences between the groups. A two-tailed *P*-value less of < 0.05 was considered statistically significant.

## Results

### Fbn2 recovered the rod b-wave amplitude

**Figure 2:**
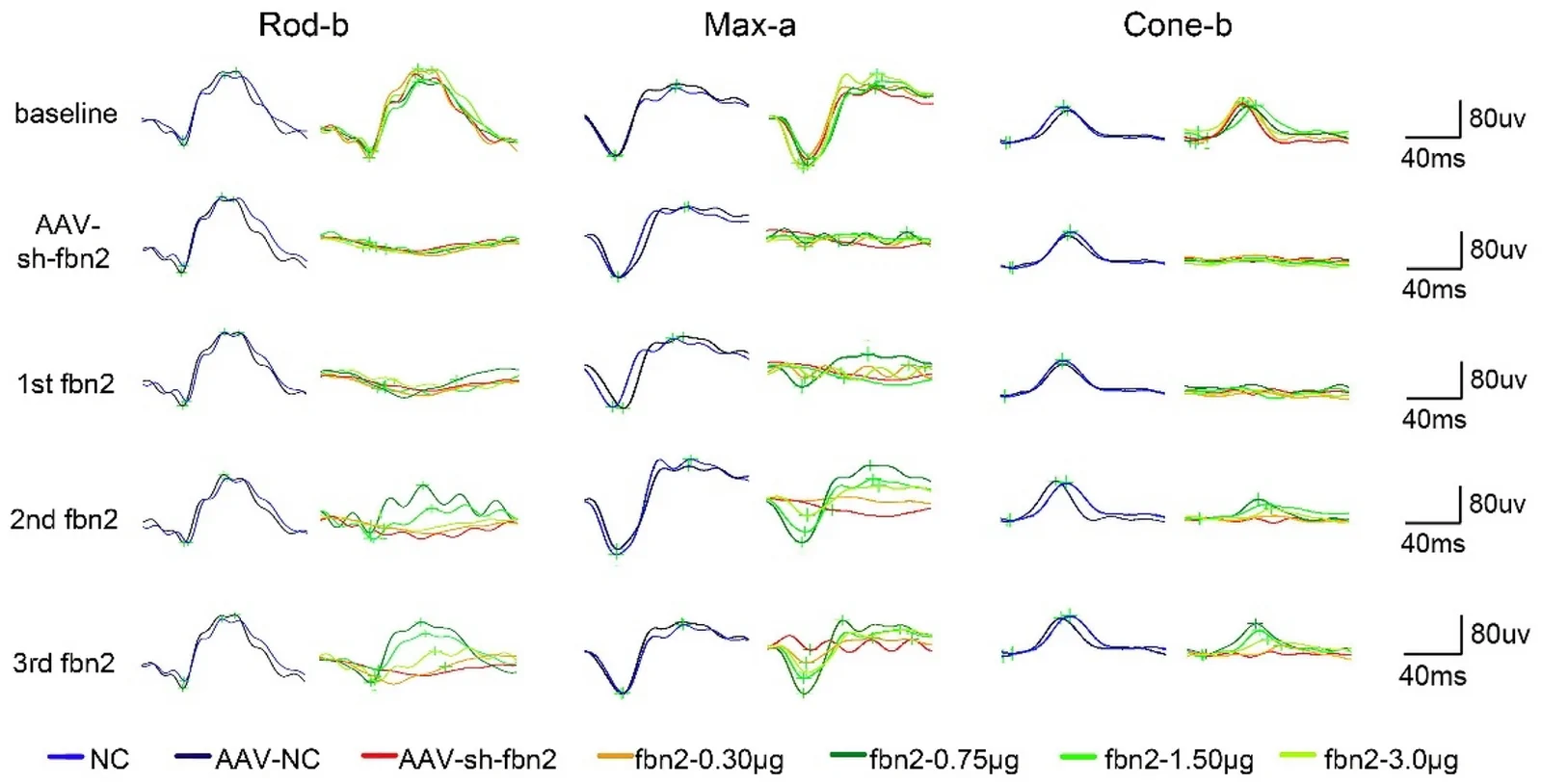
Electroretinographical analysis after intravitreal injection with empty carrier, AAV-sh-fbn2 or Fbn2 recombinant protein. NC group: Animals without intervention. AAV-NC group: Animals with an intravitreal injection of AAV empty vector and without any further treatment. AAV-sh-fbn2 group: Animals with an intravitreal injection of AAV-sh-fbn2 and without any further treatment. Fbn2-0.30 μg group: Animals with an intravitreal injection of AAV-sh-fbn2, followed by an intravitreal injection of fbn2 recombinant protein in a dose of 0.30 μg. Fbn2-0.75 μg group: Animals with an intravitreal injection of AAV-sh-fbn2, followed by an intravitreal injection of fbn2 recombinant protein in a dose of 0.75 μg. Fbn2-1.5 μg group: Animals with an intravitreal injection of AAV-sh-fbn2, followed by an intravitreal injection of fbn2 recombinant protein in a dose of 1.50 μg. Fbn2-3.0 μg group: Animals with an intravitreal injection of AAV-sh-fbn2, followed by an intravitreal injection of fbn2 recombinant protein in a dose of 3.00 μg.

All groups of animals did not differ significantly in the mean amplitude of the rod b-wave amplitude at baseline (all *P* > 0.05) (Table 1) (Fig. 2). At 2 weeks after baseline, the mean amplitude of rod b-wave amplitude was significantly lower in the eyes with intravitreal injections of AAV-sh-fbn2 (group #3–7) than in the eyes with intravitreal injections of AAV empty vector (group #2) (all *P* < 0.001) (Table 1) (Fig. 2). After the three intravitreal injections of fbn2 recombinant protein had been carried out, the mean rod b-wave amplitude was significantly higher in eyes with intravitreal injections of fbn2 recombinant protein in doses of 0.75 μg, 1.5 μg and 3.0 μg (group #5, group #6 and group #7) than in the eyes with intravitreal injections of AAV-sh-fbn2 only or in eyes without any further injections (group #3) (118.33 ± 16.42 μV vs. 26.25 ± 6.88 μV; *P* < 0.001; 77.00 ± 8.51 μV vs. 26.25 ± 6.88 μV; *P* = 0.02; 58.88 ± 9.32 μV vs. 26.25 ± 6.88 μV; *P* = 0.03; respectively). The rod b-wave amplitude did not differ significantly between eyes with three intravitreal injections of fbn2 recombinant protein in a dose of 0.75 μg (group #5), eyes without any intervention (group #1), and eyes with intravitreal injections of AAV empty vector and without any further treatment (group #2) (Table 1) (Fig. 2).

**Table 1:** Retinal electroretinogram measurements (mean ± standard deviation).

group	n	Baseline (μV)	AAV-sh-fbn2 (μV)	1st Fbn2 (μV)	2nd Fbn2(μV)	3rd Fbn2(μV)
Rod-b						
NC	9	140.40 ± 18.79	139.78 ± 22.66	130.97 ± 16.09	135.29 ± 19.72	145.38 ± 24.31
AAV-NC	9	132.41 ± 17.42	137.91 ± 18.93	142.91 ± 20.44	139.16 ± 15.09	134.91 ± 19.73
AAV-sh-fbn2	9	145.35 ± 15.80	25.08 ± 4.21**	22.28 ± 4.33**	20.96 ± 4.59**	26.25 ± 6.88**
Fbn2-0.30 μg	9	135.22 ± 18.39	23.02 ± 5.78**	26.24 ± 3.80**	28.97 ± 7.00**	31.75 ± 4.79**
Fbn2-0.75 μg	9	142.97 ± 25.58	20. 67 ± 2.23**	32.95 ± 5.67*	76.33 ± 10.46*^#^	118.33 ± 16.42^#^
Fbn2-1.5 μg	9	130.14 ± 19.36	24.45 ± 4.27**	28.38 ± 4.29**	39.79 ± 6.17*	77.00 ± 8.51*^#^
Fbn2-3.0 μg	9	148.35 ± 21.03	19.11 ± 3.78**	25.55 ± 1.75**	30.40 ± 4.42*	58.88 ± 9.32*^#^
Max-a						
NC	9	− 101.97 ± 18.67	− 116.45 ± 17.44	− 120.45 ± 17.33	− 106.45 ± 18.68	− 119.5 ± 20.38
AAV-NC	9	− 123.85 ± 15.41	− 114.07 ± 22.73	− 119.05 ± 15.85	− 108.29 ± 17.42	− 111.05 ± 16.71
AAV-sh-fbn2	9	− 117.26 ± 17.83	− 12.90 ± 2.84**	− 14.40 ± 3.33**	− 19.82 ± 3.85**	− 16.17 ± 3.66**
Fbn2-0.30 μg	9	− 110.77 ± 18.63	− 14.19 ± 2.67**	− 19.49 ± 2.38**	− 23.04 ± 6.67**	− 28.72 ± 4.33**
Fbn2-0.75 μg	9	− 115.7 ± 17.89	− 19.65 ± 2.35**	− 25.65 ± 5.22**	− 67.95 ± 9.50*^#^	− 99.44 ± 12.42^#^
Fbn2-1.5 μg	9	− 119.97 ± 12.46	− 17.90 ± 2.79**	− 22.53 ± 4.67**	− 29.24 ± 6.03*	− 65.27 ± 8.67*^#^
Fbn2-3.0 μg	9	− 114.26 ± 19.33	− 14.07 ± 3.07**	− 16.90 ± 3.64**	− 24.54 ± 5.47*	− 30.57 ± 5.88*^#^
Cone-b						
NC	9	78.33 ± 8.03	70.21 ± 10.17	75.21 ± 9.47	67.22 ± 8.99	71.31 ± 9.03
AAV-NC	9	70.61 ± 9.64	66.56 ± 11.33	60.77 ± 9.33	72.23 ± 6.65	70.03 ± 11.46
AAV-sh-fbn2	9	63.88 ± 9.59	3.89 ± 2.82**	3.03 ± 2.02**	3.77 ± 2.97**	3.07 ± 2.11**
Fbn2-0.30 μg	9	72.46 ± 10.33	4.27 ± 2.63**	3.53 ± 2.56**	7.67 ± 3.22**	8.55 ± 5.42**
Fbn2-0.75 μg	9	78.53 ± 10.67	3.03 ± 3.44**	6.21 ± 2.67	31.18 ± 8.48*^#^	58.61 ± 10.67^#^
Fbn2-1.5 μg	9	61.61 ± 11.44	2.88 ± 2.51**	4.44 ± 3.94	13.86 ± 4.03*	38.57 ± 8.03*^#^
Fbn2-3.0 μg	9	65.88 ± 9.82	3.27 ± 1.67**	3.99 ± 1.95**	8.05 ± 3.37*	17.69 ± 4.45*^#^

Rod-b, Rod b-wave amplitude (dark adaptation 0.01 ERG). Max-a, the maximal a-wave (dark adaptation 3.0 ERG). Cone-b, Cone b-wave amplitude (light adaptation 3.0 ERG).

NC, Animals without intervention. AAV-NC group, Animals with an intravitreal injection of AAV empty vector and without any further treatment. AAV-sh-fbn2, Animals with an intravitreal injection of AAV-sh-fbn2 and without any further treatment. Fbn2-0.30 μg, Animals with an intravitreal injection of AAV-sh-fbn2, followed by an intravitreal injection of fbn2 recombinant protein in a dose of 0.30 μg. Fbn2-0.75 μg, Animals with an intravitreal injection of AAV-sh-fbn2, followed by an intravitreal injection of fbn2 recombinant protein in a dose of 0.75 μg. Fbn2-1.5 μg, Animals with an intravitreal injection of AAV-sh-fbn2, followed by an intravitreal injection of fbn2 recombinant protein in a dose of 1.50 μg. Fbn2-3.0 μg, Animals with an intravitreal injection of AAV-sh-fbn2, followed by an intravitreal injection of fbn2 recombinant protein in a dose of 3.00 μg.

*P < 0.05 compared with the AAV-NC group; **P < 0.001 compared with the AAV-NC group; ^#^*P* < 0.05 compared with the AAV-sh-fbn2 group; ^##^*P* < 0.001 compared with the AAV-sh-fbn2 group.

### Fbn2 recovered the maximal (Max) a-wave amplitude

All groups did not differ significantly in the mean amplitude of Max a-wave at baseline (all *P* > 0.05) (Table 1) (Fig. 2). At 2 weeks after baseline, the mean amplitude of Max a-wave was significantly lower in eyes with the intravitreal injection of AAV-sh-fbn2 (groups #3–7) than in the eyes with the intravitreal injection of an AAV-empty vector only (group #2) (all *P* < 0.001). After three intravitreal injections of the *fbn2* recombinant protein, the mean amplitude of Max a-wave was significantly higher in the eyes with the intravitreal injection of *fbn2* recombinant protein applied in doses of 0.75 μg, 1.5 μg and 3.0 μg (groups #5, #6 and #7) than in the eyes with an intravitreal injection of AAV-sh-fbn2 and without any further injections (group #3) (− 99.44 ± 12.42 μV vs. − 16.17 ± 3.66 μV; *P* < 0.001; − 65.27 ± 8.67 μV vs. − 16.17 ± 3.66 μV; *P* = 0.02; − 30.57 ± 5.88 μV vs. − 16.17 ± 3.66 μV; *P* = 0.03). The Max a-wave amplitude did not differ significantly between the eyes with three intravitreal injections of *fbn2* recombinant protein in a dose of 0.75 μg (group #5) as compared with eyes without any intervention (group #1) or as compared with the eyes with intravitreal injection of AAV empty vector and without any further injections (group #2) (Table 1) (Fig. 2).

### Fbn2 recovered the cone b-wave amplitude

All groups did not vary significantly in cone b-wave amplitude at baseline (all *P* > 0.05) (Table 1) (Fig. 2). At 2 weeks after baseline, the mean amplitude of the cone b-wave was significantly lower in eyes with the intravitreal injection of AAV-sh-fbn2 (groups #3–7) than in the eyes with the intravitreal injection of the AAV-empty vector (group #2) (all *P* < 0.001). After three intravitreal injections of *fbn2* recombinant protein, the mean amplitude of the cone b-wave was significantly higher in the eyes with the intravitreal injection of the *fbn2* recombinant protein applied in doses of 0.75 μg, 1.5 μg and 3.0 μg (groups #5, #6 and #7) than in the eyes with intravitreal injection of AAV-sh-fbn2 and without any further treatment (group #3) (58.61 ± 10.67 μV vs. 3.07 ± 2.11 μV; *P* = 0.01; 38.57 ± 8.03 μV vs. 3.07 ± 2.11 μV; *P* = 0.02; 17.69 ± 4.45 μV vs. 3.07 ± 2.11 μV; *P* = 0.03). The cone b-wave amplitude did not vary significantly between eyes with three intravitreal injections of fbn2 recombinant protein in a dose of 0.75 μg (group #5) as compared with eyes without any intervention (group #1) or compared with eyes with intravitreal injection of the AAV empty vector and without any further treatment (group2) (Table 1) (Fig. 2).

### Alleviation of fundus lesions

**Figure 3:**
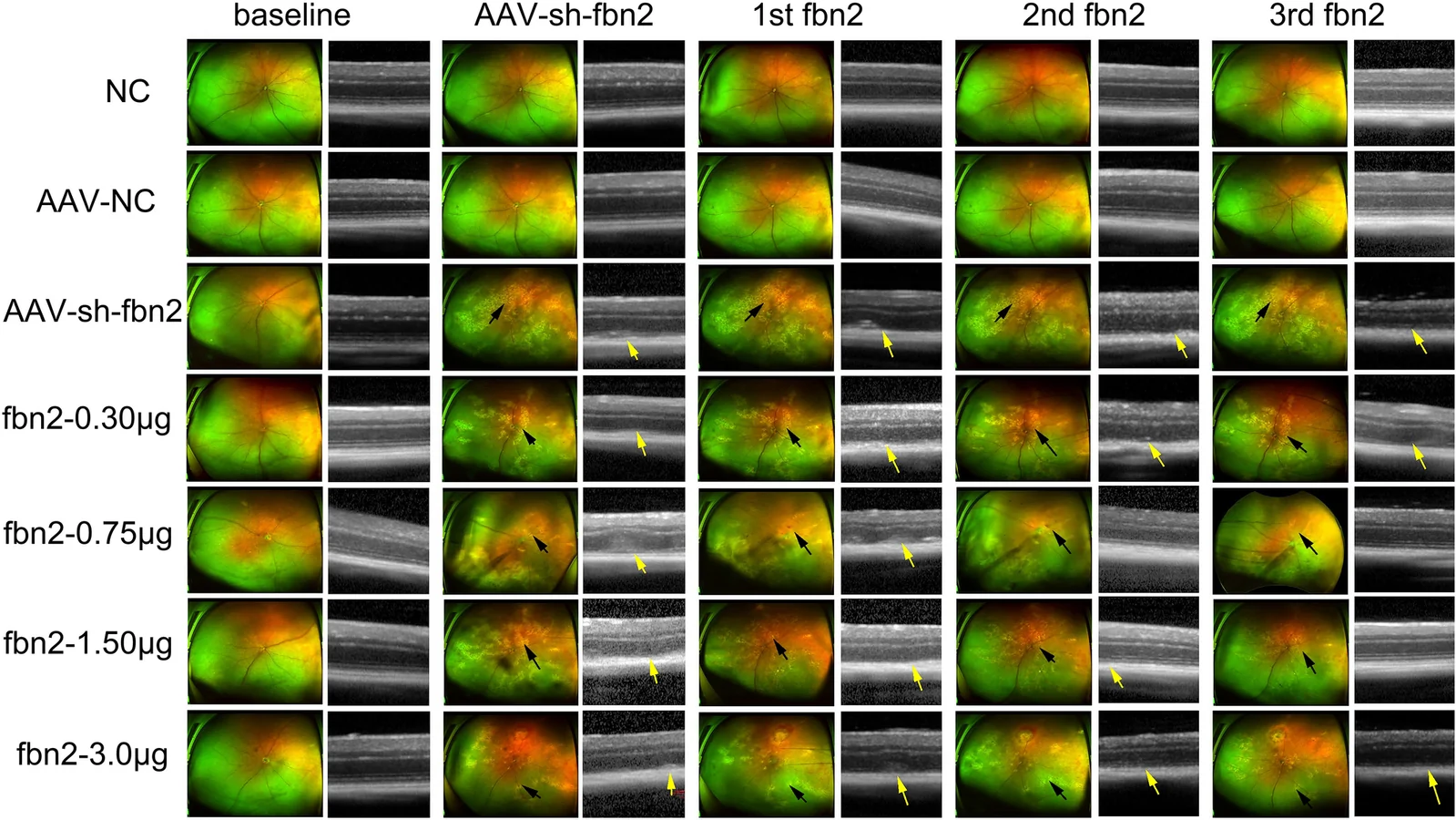
Fundus retinal imaging captured by SLO and OCT in NC, AAV-NC, AAV-sh-fbn2, Fbn2-0.30 μg, Fbn2-0.75 μg, Fbn2-1.50 μg and Fbn2-3.0 μg group mice. SLO showed irregular yellow-white exudates in the retina (Black arrow). OCT shows that light reflection in each layer of the retina becomes more irregular and reflectance decreases (Yellow arrow). NC group: Animals without intervention. AAV-NC group: Animals with an intravitreal injection of AAV empty vector and without any further treatment. AAV-sh-fbn2 group: Animals with an intravitreal injection of AAV-sh-fbn2 and without any further treatment. Fbn2-0.30 μg group: Animals with an intravitreal injection of AAV-sh-fbn2, followed by an intravitreal injection of fbn2 recombinant protein in a dose of 0.30 μg. Fbn2-0.75 μg group: Animals with an intravitreal injection of AAV-sh-fbn2, followed by an intravitreal injection of fbn2 recombinant protein in a dose of 0.75 μg. Fbn2-1.5 μg group: Animals with an intravitreal injection of AAV-sh-fbn2, followed by an intravitreal injection of fbn2 recombinant protein in a dose of 1.50 μg. Fbn2-3.0 μg group: Animals with an intravitreal injection of AAV-sh-fbn2, followed by an intravitreal injection of fbn2 recombinant protein in a dose of 3.00 μg.

Eyes with the intravitreal injections of an AAV-empty vector and without undergoing any further procedures (group #1) showed a normal fundus upon ophthalmoscopy as assessed by fundus photography, and upon OCT imaging. The eyes with the intravitreal injection of AAV-sh-fbn2 and without any further treatment (group #3) showed multifocal, small to medium sized lesions at the layer of the retinal photoreceptors / retinal pigment epithelium, irregular yellowish white exudations in the retina, localized irregular reflections in the RPE layer, without collateral edema, and collateral tissue destruction and without involvement of the inner retinal layers (Fig. 3). Compared with the eyes of group #3, the eyes with intravitreal injections of *fbn2* recombinant protein (group #5–6) showed an improvement in fundus exudates, and the OCT revealed that the light reflex of each layer of retina became more regular and the light reflectivity decreased. As compared with group #5 and group #6, there was no significant improvement in fundus exudation and retinal structure in group #4 and group #7. In group #1 and group #2, the ocular fundus remained normal (Fig. 3).

### Fbn2 increased the retinal thickness

**Figure 4:**
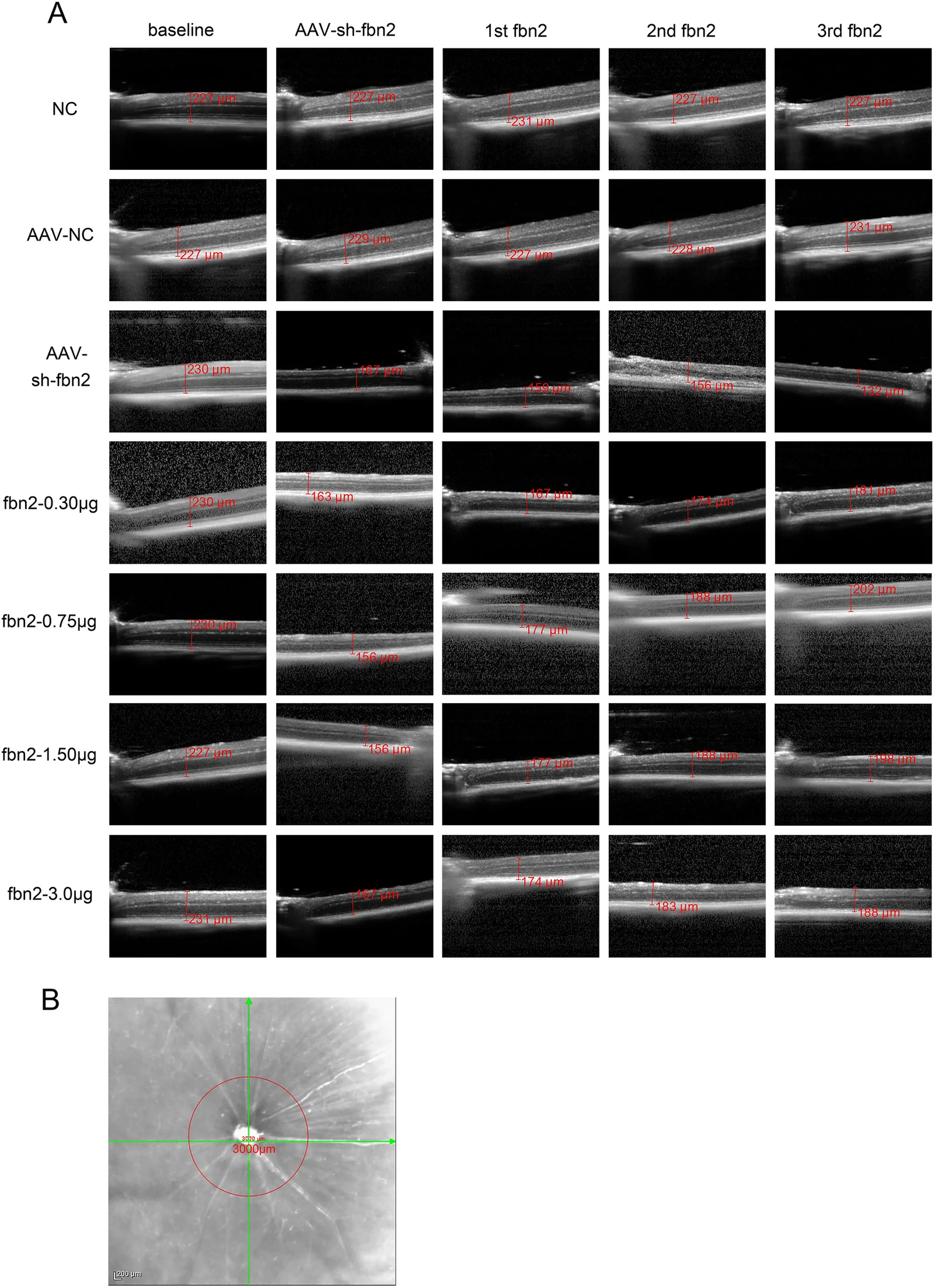
Retinal thickness imaging captured by OCT in NC, AAV-NC, AAV-sh-fbn2, Fbn2-0.30 μg, Fbn2-0.75 μg, Fbn2-1.50 μg and Fbn2-3.0 μg group mice. (**A**). Retinal thickness was measured at 3000 μm from optic disc (inferior, nasal, temporal, and superior) (**B**).NC group: Animals without intervention. AAV-NC group: Animals with an intravitreal injection of AAV empty vector and without any further treatment. AAV-sh-fbn2 group: Animals with an intravitreal injection of AAV-sh-fbn2 and without any further treatment. Fbn2-0.30 μg group: Animals with an intravitreal injection of AAV-sh-fbn2, followed by an intravitreal injection of fbn2 recombinant protein in a dose of 0.30 μg. Fbn2-0.75 μg group: Animals with an intravitreal injection of AAV-sh-fbn2, followed by an intravitreal injection of fbn2 recombinant protein in a dose of 0.75 μg. Fbn2-1.5 μg group: Animals with an intravitreal injection of AAV-sh-fbn2, followed by an intravitreal injection of fbn2 recombinant protein in a dose of 1.50 μg. Fbn2-3.0 μg group: Animals with an intravitreal injection of AAV-sh-fbn2, followed by an intravitreal injection of fbn2 recombinant protein in a dose of 3.00 μg.

Mean retinal thickness did not differ significantly between the groups at baseline (all *P* > 0.05) (Table 2) (Fig. 4). At 2 weeks after baseline, the mean retinal thickness was significantly lower in the eyes with the intravitreal injection of AAV-sh-fbn2 (groups #3–7) than in the eyes with the intravitreal injection of the AAV-empty vector (group #2) (all *P* < 0.001). After three intravitreal injections of the *fbn2* recombinant protein, the mean retinal thickness was significantly larger in the eyes with the intravitreal injection of the *fbn2* recombinant protein applied in doses of 0.75 μg, 1.5 μg and 3.0 μg (groups #5, #6 and #7) than in the eyes with the intravitreal injection of AAV-sh-fbn2 and without any further procedures (group #3) (199.02 ± 13.22 μm vs. 144.02 ± 22.88 μm, *P* = 0.02; and 190.26 ± 12.04 μm vs. 144.02 ± 22.88 μm, *P* = 0.02). The mean retinal thickness did not vary significantly between eyes with three intravitreal injections of *fbn2* recombinant protein in a dose of 0.75 μg (group #5) and the eyes without any intervention (group #1) or the eyes with the intravitreal injection of the AAV-empty vector and without any further treatment (group #2) (Table 2) (Fig. 4).

**Table 2:** Retina thickness measurements (mean ± standard deviation).

group	n	Baseline (μm)	AAV-sh-fbn2(μm)	1st Fbn2(μm)	2nd Fbn2(μm)	3rd Fbn2(μm)
NC	9	227.12 ± 5.12	228.33 ± 3.01	227.40 ± 6.93	228.09 ± 3.01	230.75 ± 2.67
AAV-NC	9	227.08 ± 6.23	227.01 ± 4.99	228.34 ± 4.75	227.20 ± 4.99	229.33 ± 3.67
AAV-sh-fbn2	9	228.09 ± 4.16	160.45 ± 12.80**	159.51 ± 17.04**	155.55 ± 26.90**	144.02 ± 22.88**
Fbn2-0.30 μg	9	230.11 ± 3.02	162.46 ± 12.36**	164.08 ± 13.20**	167.15 ± 13.17**	178.31 ± 15.06**
Fbn2-0.75 μg	9	229.95 ± 3.11	161.44 ± 8.48**	179.05 ± 9.06*	189.85 ± 13.33*^#^	199.02 ± 13.22^#^
Fbn2-1.5 μg	9	228.02 ± 2.98	161.66 ± 12.74**	171.22 ± 12.72*	179.47 ± 9.41*	190.26 ± 12.04*^#^
Fbn2-3.0 μg	9	227.85 ± 2.60	164.47 ± 10.95**	169.77 ± 12.38**	171.04 ± 11.93**	183.64 ± 19.33*

NC group, Animals without intervention. AAV-NC group, Animals with an intravitreal injection of AAV empty vector and without any further treatment. AAV-sh-fbn2 group, Animals with an intravitreal injection of AAV-sh-fbn2 and without any further treatment. Fbn2-0.30 μg group, Animals with an intravitreal injection of AAV-sh-fbn2, followed by an intravitreal injection of fbn2 recombinant protein in a dose of 0.30 μg. Fbn2-0.75 μg group, Animals with an intravitreal injection of AAV-sh-fbn2, followed by an intravitreal injection of fbn2 recombinant protein in a dose of 0.75 μg. Fbn2-1.5 μg group, Animals with an intravitreal injection of AAV-sh-fbn2, followed by an intravitreal injection of fbn2 recombinant protein in a dose of 1.50 μg. Fbn2-3.0 μg group, Animals with an intravitreal injection of AAV-sh-fbn2, followed by an intravitreal injection of fbn2 recombinant protein in a dose of 3.00 μg.

*P < 0.05 compared with the AAV-NC group; **P < 0.001 compared with the AAV-NC group; ^#^*P* < 0.05 compared with the AAV-sh-fbn2 group; ^##^*P* < 0.001 compared with the AAV-sh-fbn2 group.

### Fbn2 suppressed the elongation of axial length

**Figure 5:**
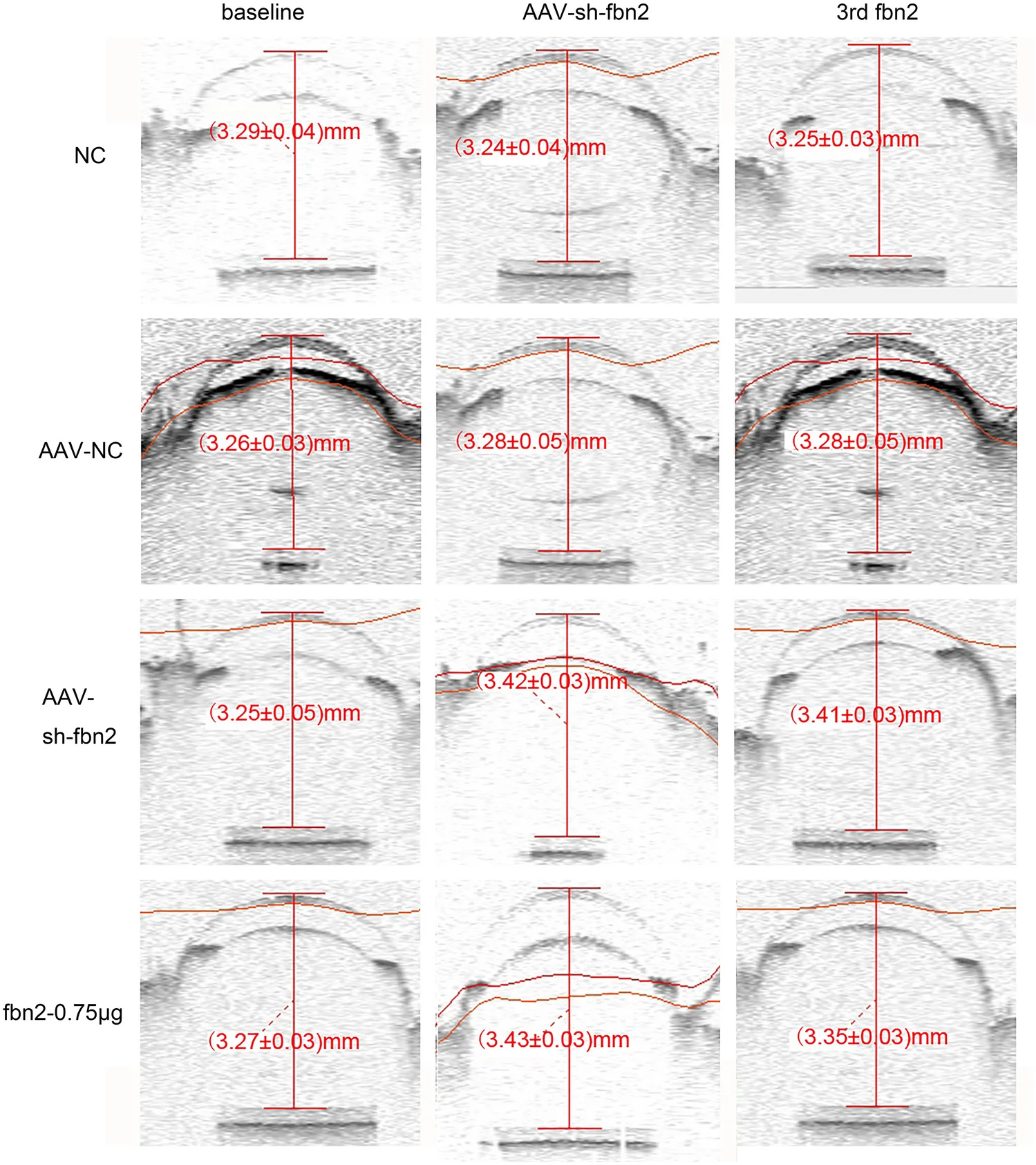
Axial length measured by OCT. NC group: Animals without intervention. AAV-NC group: Animals with an intravitreal injection of AAV empty vector and without any further treatment. AAV-sh-fbn2 group: Animals with an intravitreal injection of AAV-sh-fbn2 and without any further treatment. Fbn2-0.30 μg group: Animals with an intravitreal injection of AAV-sh-fbn2, followed by an intravitreal injection of fbn2 recombinant protein in a dose of 0.30 μg. Fbn2-0.75 μg group: Animals with an intravitreal injection of AAV-sh-fbn2, followed by an intravitreal injection of fbn2 recombinant protein in a dose of 0.75 μg. Fbn2-1.5 μg group: Animals with an intravitreal injection of AAV-sh-fbn2, followed by an intravitreal injection of fbn2 recombinant protein in a dose of 1.50 μg. Fbn2-3.0 μg group: Animals with an intravitreal injection of AAV-sh-fbn2, followed by an intravitreal injection of fbn2 recombinant protein in a dose of 3.00 μg. NC group: Animals without intervention. AAV-NC group: Animals with an intravitreal injection of AAV empty vector and without any further treatment. AAV-sh-fbn2 group: Animals with an intravitreal injection of AAV-sh-fbn2 and without any further treatment. Fbn2-0.30 μg group: Animals with an intravitreal injection of AAV-sh-fbn2, followed by an intravitreal injection of fbn2 recombinant protein in a dose of 0.30 μg. Fbn2-0.75 μg group: Animals with an intravitreal injection of AAV-sh-fbn2, followed by an intravitreal injection of fbn2 recombinant protein in a dose of 0.75 μg. Fbn2-1.5 μg group: Animals with an intravitreal injection of AAV-sh-fbn2, followed by an intravitreal injection of fbn2 recombinant protein in a dose of 1.50 μg. Fbn2-3.0 μg group: Animals with an intravitreal injection of AAV-sh-fbn2, followed by an intravitreal injection of fbn2 recombinant protein in a dose of 3.00 μg.

Mean axial length did not vary significantly between the groups at baseline (all *P* > 0.05) (Fig. 5). At 2 weeks after baseline, mean axial length was significantly shorter in the eyes with the intravitreal injection of AAV-sh-fbn2 (group #3 and group #5) than in the eyes with the intravitreal injection of the AAV-empty vector (group #2) (*P* < 0.001). After three intravitreal injections of the *fbn2* recombinant protein, the mean axial length was significantly shorter in the eyes with the intravitreal injection of the *fbn2* recombinant protein applied in a dose of 0.75 μg (group #5) than in the eyes with the intravitreal injection of AAV-sh-fbn2 and without any further procedures (group #3) (3.41 ± 0.03 mm vs. 3.34 ± 0.03 mm, *P* = 0.04) (Fig. 5). Axial length in the eyes with the intravitreal injection of the *fbn2* recombinant protein in a dose of 0.75 μg (group #5) was significantly longer than that of eyes without any intervention (group #1), and than that of the eyes with an intravitreal injection of the AAV-empty vector and without any further treatments (group2). The OCT examination did not show any signs of inflammation or edema in any part or tissue of the eyes, neither in the eyes with the fbn2-shRNA injections or in the eyes with the *fbn2*-recombinant protein injection.

### Endogenous mRNA expression of FBN2, TGF-β1 and LTBP-1 in the retina

**Figure 6:**
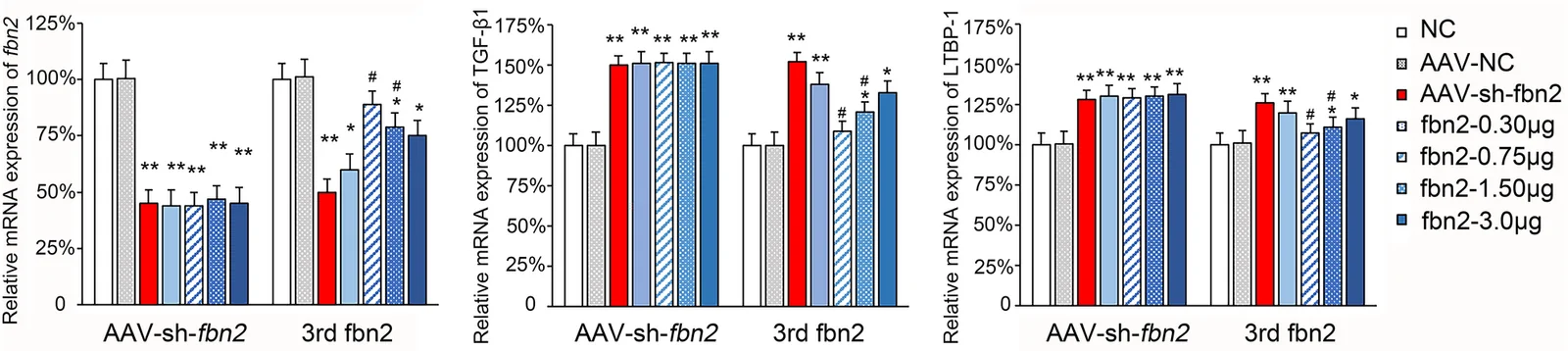
Measurement of fbn2, Tgf-β1 and LTBP-1 expression at mRNA levels in the retina of mice in NC, AAV-NC, AAV-sh-fbn2, Fbn2-0.30 μg, Fbn2-0.75 μg, Fbn2-1.50 μg and Fbn2-3.0 μg groups (all groups n = 9). Data are shown as mean ± SEM. *P < 0.05 all other groups versus AAV-NC group; **P < 0.001 all other groups versus AAV-NC group; #*P* < 0.05 all other groups versus AAV-sh-fbn2 group NC group: Animals without intervention. AAV-NC group: Animals with an intravitreal injection of AAV empty vector and without any further treatment. AAV-sh-fbn2 group: Animals with an intravitreal injection of AAV-sh-fbn2 and without any further treatment. Fbn2-0.30 μg group: Animals with an intravitreal injection of AAV-sh-fbn2, followed by an intravitreal injection of fbn2 recombinant protein in a dose of 0.30 μg. Fbn2-0.75 μg group: Animals with an intravitreal injection of AAV-sh-fbn2, followed by an intravitreal injection of fbn2 recombinant protein in a dose of 0.75 μg. Fbn2-1.5 μg group: Animals with an intravitreal injection of AAV-sh-fbn2, followed by an intravitreal injection of fbn2 recombinant protein in a dose of 1.50 μg. Fbn2-3.0 μg group: Animals with an intravitreal injection of AAV-sh-fbn2, followed by an intravitreal injection of fbn2 recombinant protein in a dose of 3.00 μg.

Two weeks after the intravitreal injection of AAV-sh-fbn2, the mRNA expression of endogenous *fbn2* was significantly lower and the mRNA expression of endogenous of TGF-β1 and LTBP-1 was significantly higher in the eyes with the intravitreal injection of AAV-sh-fbn2 (groups #3–7) than in the eyes with the intravitreal injection of the AAV-empty vector (group #2) (all *P* < 0.05) (Fig. 6). After three intravitreal injections of the *fbn2* recombinant protein, the mRNA expression of endogenous *fbn2* was significantly higher and the mRNA expression of endogenous TGF-β1 and LTBP-1 was significantly lower in the eyes with the intravitreal injections of the *fbn2* recombinant protein applied in doses of 0.75 μg, 1.5 μg and 3.0 μg (groups #5, #6 and #7) than in the eyes with the intravitreal injection of AAV-sh-fbn2 and without any further procedures (group #3) (all *P* < 0.05) (Fig. 6). The mRNA expression of endogenous TGF-β1 and LTBP-1 in the eyes with three intravitreal injections of the *fbn2* recombinant protein in a dose of 0.75 μg (group #5) did not differ significantly from the expression in the eyes without any intervention (group #1) and the eyes with the intravitreal injection of the AAV-empty vector and without any further procedures (group #2).

### Protein expression of fbn2, TGF-β1 and LTBP-1 in the retina

**Figure 7:**
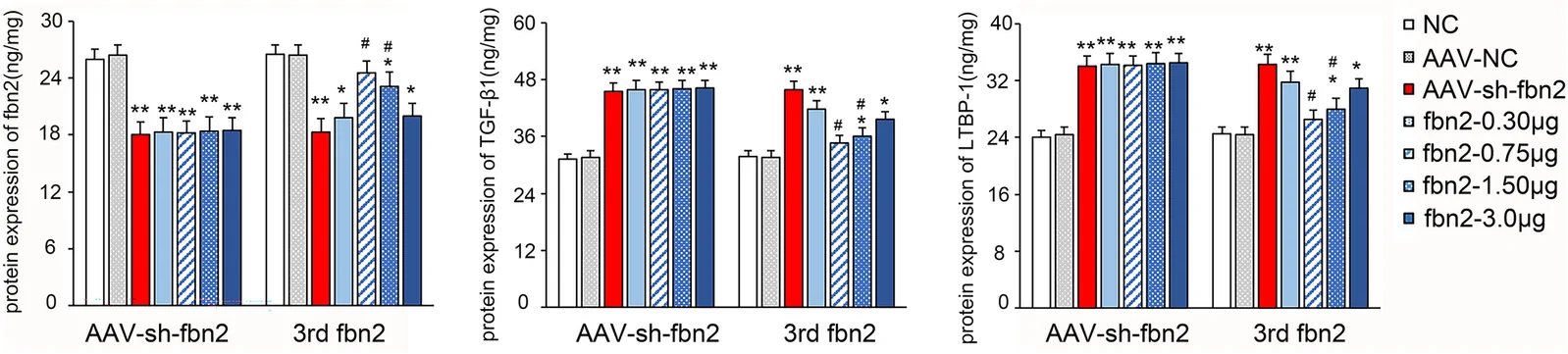
Elisa measurement of Fbn2, Tgf-β1 and LTBP-1 expression at protein levels in retina of mice in NC, AAV-NC, AAV-sh-fbn2, Fbn2-0.30 μg, Fbn2-0.75 μg, Fbn2-1.50 μg and Fbn2-3.0 μg group (all groups n = 9). Data are shown as mean ± SEM. *P < 0.05 all other groups versus AAV-NC group; **P < 0.001 all other groups versus AAV-NC group; ^#^*P* < 0.05 all other groups versus AAV-sh-fbn2 group. NC group: Animals without intervention. AAV-NC group: Animals with an intravitreal injection of AAV empty vector and without any further treatment. AAV-sh-fbn2 group: Animals with an intravitreal injection of AAV-sh-fbn2 and without any further treatment. Fbn2-0.30 μg group: Animals with an intravitreal injection of AAV-sh-fbn2, followed by an intravitreal injection of fbn2 recombinant protein in a dose of 0.30 μg. Fbn2-0.75 μg group: Animals with an intravitreal injection of AAV-sh-fbn2, followed by an intravitreal injection of fbn2 recombinant protein in a dose of 0.75 μg. Fbn2-1.5 μg group: Animals with an intravitreal injection of AAV-sh-fbn2, followed by an intravitreal injection of fbn2 recombinant protein in a dose of 1.50 μg. Fbn2-3.0 μg group: Animals with an intravitreal injection of AAV-sh-fbn2, followed by an intravitreal injection of fbn2 recombinant protein in a dose of 3.00 μg.

**Figure 8:**
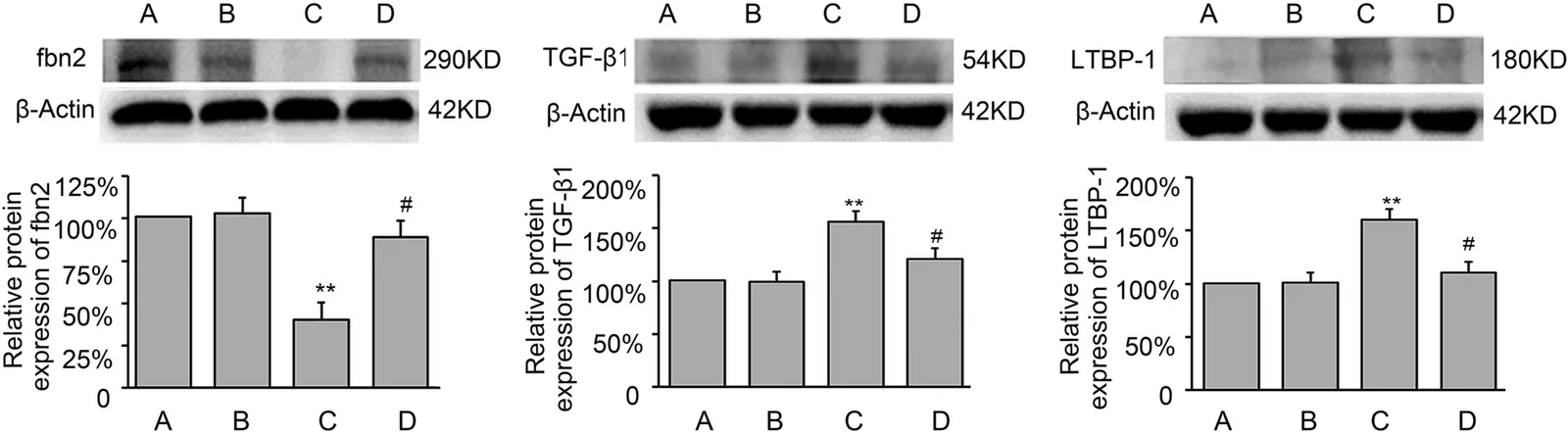
Measurement of fbn2, Tgf-β1 and LTBP-1 expression at protein levels using Western blot in the retina of mice in NC, AAV-NC, AAV-sh-fbn2 and Fbn2-0.75 μg group (all groups n = 9). Data are shown as mean ± SEM. **P < 0.001 all other groups versus AAV-NC group; ^#^*P* < 0.05 all other groups versus AAV-sh-fbn2 group. Original blots are presented in Supplementary Fig. 2–5. A: NC group, B: AAV-NC group, C: AAV-sh-fbn2 group, D: Fbn2-0.75 μg group. (**P < 0.001 compared with the AAV-NC group; ^#^*P* < 0.05 compared with the AAV-sh-fbn2 group). NC group: Animals without intervention. AAV-NC group: Animals with an intravitreal injection of AAV empty vector and without any further treatment. AAV-sh-fbn2 group: Animals with an intravitreal injection of AAV-sh-fbn2 and without any further treatment. Fbn2-0.30 μg group: Animals with an intravitreal injection of AAV-sh-fbn2, followed by an intravitreal injection of fbn2 recombinant protein in a dose of 0.30 μg. Fbn2-0.75 μg group: Animals with an intravitreal injection of AAV-sh-fbn2, followed by an intravitreal injection of fbn2 recombinant protein in a dose of 0.75 μg. Fbn2-1.5 μg group: Animals with an intravitreal injection of AAV-sh-fbn2, followed by an intravitreal injection of fbn2 recombinant protein in a dose of 1.50 μg. Fbn2-3.0 μg group: Animals with an intravitreal injection of AAV-sh-fbn2, followed by an intravitreal injection of fbn2 recombinant protein in a dose of 3.00 μg.

After intravitreal injection of AAV-sh-fbn2 for 2 weeks, the protein expression of *fbn2* was significantly lower and the protein expression of TGF-β1 and LTBP-1 was significantly higher in the retina of eyes with the intravitreal injection of AAV-sh-fbn2 (groups #3–7) than in eyes with intravitreal injection of AAV-empty vector (group #2) (*P* < 0.05; Fig. 7). After three intravitreal injections of *fbn2* recombinant protein, the protein expression of *fbn2* increased significantly and the protein expression of TGF-β1 and LTBP-1 decreased significantly in the eyes with the intravitreal injection of fbn2 recombinant protein in doses of 0.75 μg, 1.5 μg and 3.0 μg (groups #5, #6 and #7) than in the retina of the eyes with the intravitreal injection of AAV-sh-fbn2 and without any treatment (group #3) (*P* < 0.05; Fig. 7). The protein levels of TGF-β1 and LTBP-1 in the retina of the eyes with three intravitreal injections of *fbn2* recombinant protein in a dose of 0.75 μg (group #5) were not significantly different from those in the retina of the eyes without any experiment (group #1) or in the retina of the eyes with the intravitreal injection of the AAV-empty vector (group2) (Fig. 8).

## Discussion

In our experimental study, the intravitreal injection of AAV-sh-fbn2 led to the development of a retinopathy characterized by fundus exudation with multifocal lesions in the deep retinal layers and retinal pigment epithelium, parallel to a reduction in the ERG amplitudes. The retinopathy showed some similarities with a macular degeneration in patients^1,10,11^. After intravitreal treatment with the *fbn2* recombinant protein, the mRNA and protein expression of *fbn2* increased in the retina, while the mRNA and protein expression of TGF-β1 and LTBP-1 decreased. In a parallel manner, the retinopathy improved. The observations may suggest that the *fbn2* recombinant protein compensated for a externally induced, genetically based *fbn2* deficiency in the retina through regulating the TGF-β1/LTBP-1 signal pathway.

Rare and common variants in FBN2 can contribute to Mendelian and complex forms of macular degeneration. A non-synonymous variant of the FBN2 gene, rs154001 (p.Val965Ile), was identified to be associated with AMD, and the non-synonymous variants of the FBN2 gene, including c.3430G-A (p. Glu1144Lys), c.3740T-C(p.Met1247Thr), c.4312G > A(p.Glu1438Lys) and c.4141C > A (p.His1381Asn) were identified in patients with EOMD^1^. It has been reported that the FBN2 protein is located in Bruch's membrane, that it is reduced in AMD eyes, and that a reduction in the expression of fbn2 in the retina was associated with the development of a retinopathy in mice^1,7^. Recombinant proteins have been considered to be therapeutic agents for the treatment of inherited diseases, however, their clinical application has been limited due to their inability to enter the targeted cells and to be functional in the intracellular site. Intravitreal injections of antibodies have been helpful to directly reach the retina and cure eye diseases clinically and experimentally, which could break the barrier for use of recombinant proteins as therapeutic agents^12^. On this basis, this study aimed to investigate the therapeutic effect of FBN2 recombinant protein on FBN2-deficient retinopathy, to provide theoretical basis for targeted intervention of gene defective retinopathy.

Our findings showed that the intravitreal injection of *fbn2* recombinant protein reversed the effect of intravitreally applied AAV-sh-fbn2, including a re-increase in the ERG amplitude and an amelioration of the induced fundus changes such as intraretinal exudations. Interestingly, the intravitreally applied AAV-sh-fbn2 led to the development of irregularities in the RPE-layer, suggesting that the fbn2 recombinant protein led to lesions in the RPE-layer, which improved after the intravitreal application of the *fbn2* recombinant protein. It may also be noted that the *fbn2* deficiency was related to axial elongation, while the axial length was significantly reduced in the eyes after the intravitreal injections of the *fbn2* recombinant protein. It might suggest that a fbn2 deficiency might also be associated with axial myopia. It concurs with previous reports that congenital contractural arachnodactyly (CCA) induced by mutations of FBN2 gene exhibited high myopia-related syndromes^13^.

The observation made in our study may have some implications. The TGF-β family, including TGF-β1, TGF-β2 and TGF-β3, is closely correlated with subretinal fibrosis^14^. FBN2 regulates the bioavailability of TGF-β in the retina of mice, and TGF-β1 is increased when the FBN2 expression is decreased^15^. Studies have revealed that TGF-β signaling plays a role in the process of the subretinal scar formation in eyes with neovascular AMD and may directly induce the proliferation of RPE cells and it may activate fibroblasts^16^. All 3 transforming growth factor beta isoforms (e.g., TGFB1; 190180) are secreted as large latent complexes that have no biologic activity. The large latent complex has 3 components: a disulfide-bonded homodimer of mature TGFB associated noncovalently with latency-associated proteins (LAPs), which are homodimers of the N-terminal fragment of precursor TGFB, and a covalently attached latent TGFB-binding protein (LTBP), such as LTBP1. LAPs are sufficient to render the mature TGFB homodimer inactive, and removal of both the LAPs and LTBP, or modulation of their interaction, is essential for the TGFB function^17^. The latter may be associated with an accumulation of extracellular collagen and fibrosis, as can be found in eyes with AMD^18^. It may suggest that *fbn2* may potentially be of interest to further explore molecular factors for treatment of *fbn2* deficiency-induced macular degeneration and other forms of proliferative macular degeneration, potentially through suppressing the expression of LTBP and TGF in the retina.

When the results of our study are discussed, its limitations have to be taken into account. First, it was an experimental study including mice, so that further studies are mandatory to examine the transferability of the finding on humans. It holds true in particular since mice, in contrast to primates, do not have a macula. Second, the model of genetically a *fbn2* deficiency-associated retinopathy may differ from the *fbn2* deficiency-related retinopathy in patients. Third, the study period was short, so long-term changes in association with the intravitreal application of the *fbn2* recombinant protein could not be examined. Fourth, the human FBN2 protein was used in this study, but the transcriptional product of FBN2 gene of mice and human share 97% homology^19^. Our study suggested that human recombinant protein pairs can improve the symptoms of fbn2-deficient retinopathy in mice, suggesting that the functional domain of fbn2 may be located in the homology of the FBN2 gene of mice and human being.

In conclusion, the intravitreal application of AAV-sh-fbn2 led to an fbn2 deficiency-related retinopathy in mice. The signs of this retinopathy could be partially reversed by the repeated intravitreal application of the *fbn2* recombinant protein.

## Supplementary Information


Supplementary Information 1.Supplementary Information 2.

## Data Availability

All data generated or analyzed during this study are included in this published article and its supplementary information files.

## Abbreviations

Fbn2, Fibrillin 2: AMD, age-related macular degeneration
EOMD: Early-onset macular degeneration
AAV: Adeno-associated virus
TGF-β1: Transforming growth factor-beta
LTBP-1: TGF-β binding protein
ERG: Electroretinography
OCT: Optical coherence tomography
SLO: Scanning laser confocal ophthalmoscope
RGCs: Retinal ganglion cell
ELISA: Enzyme linked immunosorbent assay
RT-PCR: Reverse transcriptase polymerase chain reaction
SDS-PAGE: Sodium dodecyl sulfate polyacrylamide gel electrophoresis
Max: Maximal

## References

[1] Ratnapriya, R. *et al.* Rare and common variants in extracellular matrix gene fibrillin 2 (FBN2) are associated with macular degeneration. *Hum. Mol. Genet.* **23**(21), 5827–5837 (2014). 24899048 10.1093/hmg/ddu276 PMC4189898

[2] Sakai, L. Y., Keene, D. R. & Engvall, E. Fibrillin, a new 350-kD glycoprotein, is a component of extracellular microfibrils. *J. Cell Biol.* **103**(6 Pt 1), 2499–2509 (1986). 3536967 10.1083/jcb.103.6.2499 PMC2114568

[3] Dietz, H. C. *et al.* Marfan syndrome caused by a recurrent de novo missense mutation in the fibrillin gene. *Nature* **352**(6333), 337–339 (1991). 1852208 10.1038/352337a0

[4] Vierkotten, S., Muether, P. S. & Fauser, S. Overexpression of HTRA1 leads to ultrastructural changes in the elastic layer of Bruch’s membrane via cleavage of extracellular matrix components. *PLoS ONE* **6**(8), e22959 (2011). 21829675 10.1371/journal.pone.0022959 PMC3149070

[5] Corson, G. M., Charbonneau, N. L., Keene, D. R. & Sakai, L. Y. Differential expression of fibrillin-3 adds to microfibril variety in human and avian, but not rodent, connective tissues. *Genomics* **83**(3), 461–472 (2004). 14962672 10.1016/j.ygeno.2003.08.023

[6] Verdera, H. C., Kuranda, K. & Mingozzi, F. AAV vector immunogenicity in humans: A long journey to successful gene transfer. *Mol. Ther.* **28**(3), 723–746 (2020). 31972133 10.1016/j.ymthe.2019.12.010 PMC7054726

[7] Xu, F. *et al.* Association between anti-fibrillin-2 protein induced retinal degeneration via intravitreous delivery and activated TGF-β signalling in mice. *Clin. Exp. Pharmacol. Physiol.* **49**(5), 586–595 (2022). 35108420 10.1111/1440-1681.13631

[8] Qiu, S. *et al.* Intravitreal injection of docosahexaenoic acid attenuated photoreceptor cell injury in a NaIO3-induced age-related macular degeneration rat model. *Neurosci. Lett.* **657**, 53–61 (2017). 28751206 10.1016/j.neulet.2017.07.041

[9] Jiang, W. J. *et al.* Amphiregulin antibody and reduction of axial elongation in experimental myopia. *EBioMedicine* **17**, 134–144 (2017). 28256400 10.1016/j.ebiom.2017.02.021 PMC5360597

[10] Thomas, C. J., Mirza, R. G. & Gill, M. K. Age-related macular degeneration. *Med. Clin. N. Am.* **105**(3), 473–491 (2021). 33926642 10.1016/j.mcna.2021.01.003

[11] Fleckenstein, M. *et al.* Age-related macular degeneration. *Nat. Rev. Dis. Primer.* **7**(1), 31 (2021). 10.1038/s41572-021-00265-2 PMC12878645 33958600

[12] Yasukawa, T. *et al.* Drug delivery systems for vitreoretinal diseases. *Prog. Retin. Eye Res.* **23**, 253–281 (2004). 15177203 10.1016/j.preteyeres.2004.02.003

[13] Liu, W. *et al.* A novel FBN2 mutation in a Chinese family with congenital contractural arachnodactyly. *FEBS Open Bio* **5**, 163–166 (2015). 25834781 10.1016/j.fob.2015.02.005 PMC4359973

[14] Tosi, G. M., Orlandini, M. & Galvagni, F. The controversial role of TGF-β in neovascular age-related macular degeneration pathogenesis. *Int. J. Mol. Sci.* **19**(11), 3363 (2018). 30373226 10.3390/ijms19113363 PMC6275040

[15] Nistala, H. *et al.* Fibrillin-1 and -2 differentially modulate endogenous TGF-β and BMP bioavailability during bone formation. *J. Cell Biol.* **190**(6), 1107–1121 (2010). 20855508 10.1083/jcb.201003089 PMC3101602

[16] Schlecht, A. *et al.* Deletion of endothelial transforming growth factor-β signaling leads to choroidal neovascularization. *Am. J. Pathol.* **187**(11), 2570–2589 (2017). 28823871 10.1016/j.ajpath.2017.06.018

[17] Oklu, R. & Hesketh, R. The latent transforming growth factor beta binding protein (LTBP) family. *Biochem. J.* **352**, 601–610 (2000). 11104663 PMC1221494

[18] García-Onrubia, L. *et al.* Matrix metalloproteinases in age-related macular degeneration (AMD). *Int. J. Mol. Sci.* **21**(16), 5934 (2020). 32824762 10.3390/ijms21165934 PMC7460693

[19] Zhang, H., Hu, W. & Ramirez, F. Developmental expression of fibrillin genes suggests heterogeneity of extracellular microfibrils. *J. Cell Biol.* **129**(4), 1165–1176 (1995). 7744963 10.1083/jcb.129.4.1165 PMC2120487

