# Author Correction: Intravitreal injection of fibrillin 2 (Fbn2) recombinant protein for therapy of retinopathy in a retina-specific Fbn2 knock-down mouse model

**DOI:** 10.1038/s41598-023-47227-0

**Published:** 2023-11-15

**Authors:** Rui Xue Zhang, Ying Wen, Da Dong Guo, Fu Ru Xu, Gui Min Wang, Xing Rong Wang, Yong Wei Shi, Jie Ding, Qian Jiang, Wen Jun Jiang, Jost B. Jonas, Hong Sheng Bi

**Affiliations:** 1 Shandong University of Traditional Chinese Medicine, Jinan, China; 2 Affiliated Eye Hospital of Shandong University of Traditional Chinese Medicine, Jinan, China; 3 Shandong Provincial Key Laboratory of Integrated Traditional Chinese and Western Medicine for Prevention and Therapy of Ocular Diseases, Key Laboratory of Integrated Traditional Chinese and Western Medicine for Prevention and Therapy of Ocular Diseases in Universities of Shandong, Shandong Academy of Eye Disease Prevention and Therapy, Jinan, China; 4 Department of Ophthalmology, Medical Faculty Mannheim of the Ruprecht-Karls-University Heidelberg, Mannheim, Germany

Correction to: *Scientific Reports* https://doi.org/10.1038/s41598-023-33886-6, published online 26 April 2023

The original version of this Article contained an error in Affiliation 1, which was incorrectly given as ‘Department of Ophthalmology and Optometry, Shandong University of Traditional Chinese Medicine, Jinan, China’. The correct affiliation is listed below:

Shandong University of Traditional Chinese Medicine, Jinan, China.

The original Article has been corrected.

