# Correction: Intravitreal injection of fibrillin 2 (Fbn2) recombinant protein for therapy of retinopathy in a retina-specific Fbn2 knock-down mouse model

**DOI:** 10.1038/s41598-026-67579-7

**Published:** 2026-08-28

**Authors:** Rui Xue Zhang, Ying Wen, Da Dong Guo, Fu Ru Xu, Gui Min Wang, Xing Rong Wang, Yong Wei Shi, Jie Ding, Qian Jiang, Wen Jun Jiang, Jost B. Jonas, Hong Sheng Bi

**Affiliations:** 1 Shandong University of Traditional Chinese Medicine, Jinan, China; 2 Affiliated Eye Hospital of Shandong University of Traditional Chinese Medicine, Jinan, China; 3 Shandong Provincial Key Laboratory of Integrated Traditional Chinese and Western Medicine for Prevention and Therapy of Ocular Diseases, Key Laboratory of Integrated Traditional Chinese and Western Medicine for Prevention and Therapy of Ocular Diseases in Universities of Shandong, Shandong Academy of Eye Disease Prevention and Therapy, Jinan, China; 4 Department of Ophthalmology, Medical Faculty Mannheim of the Ruprecht-Karls-University Heidelberg, Mannheim, Germany

Correction to: *Scientific Reports* https://doi.org/10.1038/s41598-023-33886-6, published online 26 April 2023

This Article contains errors.

As a result of errors during figure assembly, several images in Figure 4 and 5 derived from the same source data. Specifically, the following images are overlapping:

- panels for fbn2-0.30 µg (2nd fbn2) and fbn2-3.0 µg (AAV-sh-fbn2) in Figure 4A,
- panels for fbn2-0.30 µg (3rd fbn2) and fbn2-1.50 µg (1st fbn2) in Figure 4A,
- panels for fbn2-0.75 µg (AAV-sh-fbn2) and fbn2-3.0 µg (2nd fbn2) in Figure 4A,
- panels for NC (AAV-sh-fbn2) and AAV-NC (AAV-sh-fbn2) in Figure 5,
- panels for AAV-NC (baseline) and AAV-NC (3rd fbn2) in Figure 5,
- and panels for fbn2-0.75 µg (baseline) and fbn2-0.75 µg (3rd fbn2) in Figure 5.

The corrected Figures 4 and 5 and their accompanying legends are shown below.

**Fig. 4:**
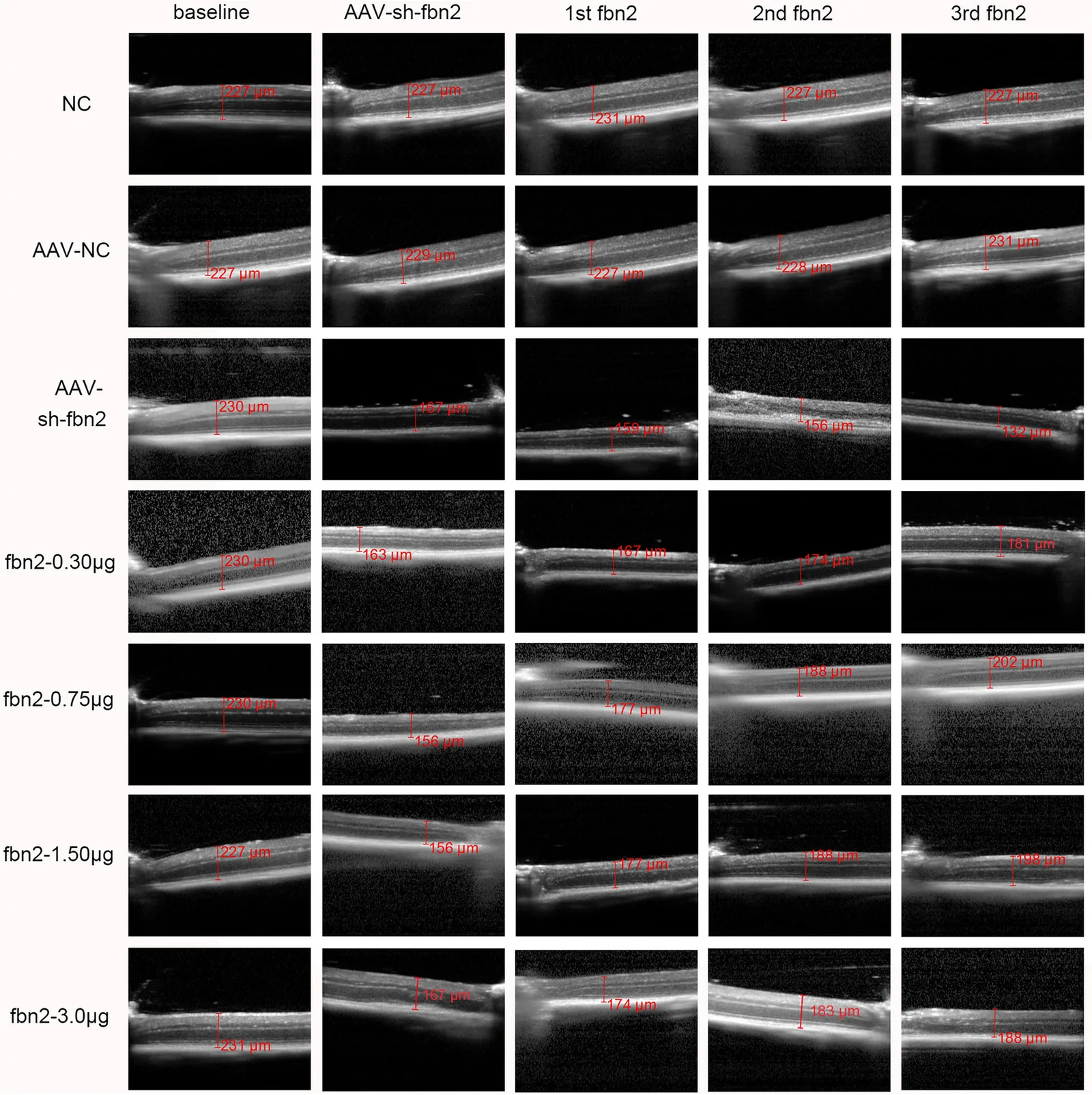
Retinal thickness imaging captured by OCT in NC, AAV-NC, AAV-sh-fbn2, Fbn2-0.30 μg, Fbn2-0.75 μg, Fbn2-1.50 μg and Fbn2-3.0 μg group mice. (**A**). Retinal thickness was measured at 3000 μm from optic disc (inferior, nasal, temporal, and superior) (**B**). NC group: Animals without intervention. AAV-NC group: Animals with an intravitreal injection of AAV empty vector and without any further treatment. AAV-sh-fbn2 group: Animals with an intravitreal injection of AAV-sh-fbn2 and without any further treatment. Fbn2-0.30 μg group: Animals with an intravitreal injection of AAV-sh-fbn2, followed by an intravitreal injection of fbn2 recombinant protein in a dose of 0.30 μg. Fbn2-0.75 μg group: Animals with an intravitreal injection of AAV-sh-fbn2, followed by an intravitreal injection of fbn2 recombinant protein in a dose of 0.75 μg. Fbn2-1.5 μg group: Animals with an intravitreal injection of AAV-sh-fbn2, followed by an intravitreal injection of fbn2 recombinant protein in a dose of 1.50 μg. Fbn2-3.0 μg group: Animals with an intravitreal injection of AAV-sh-fbn2, followed by an intravitreal injection of fbn2 recombinant protein in a dose of 3.00 μg.

**Fig. 5:**
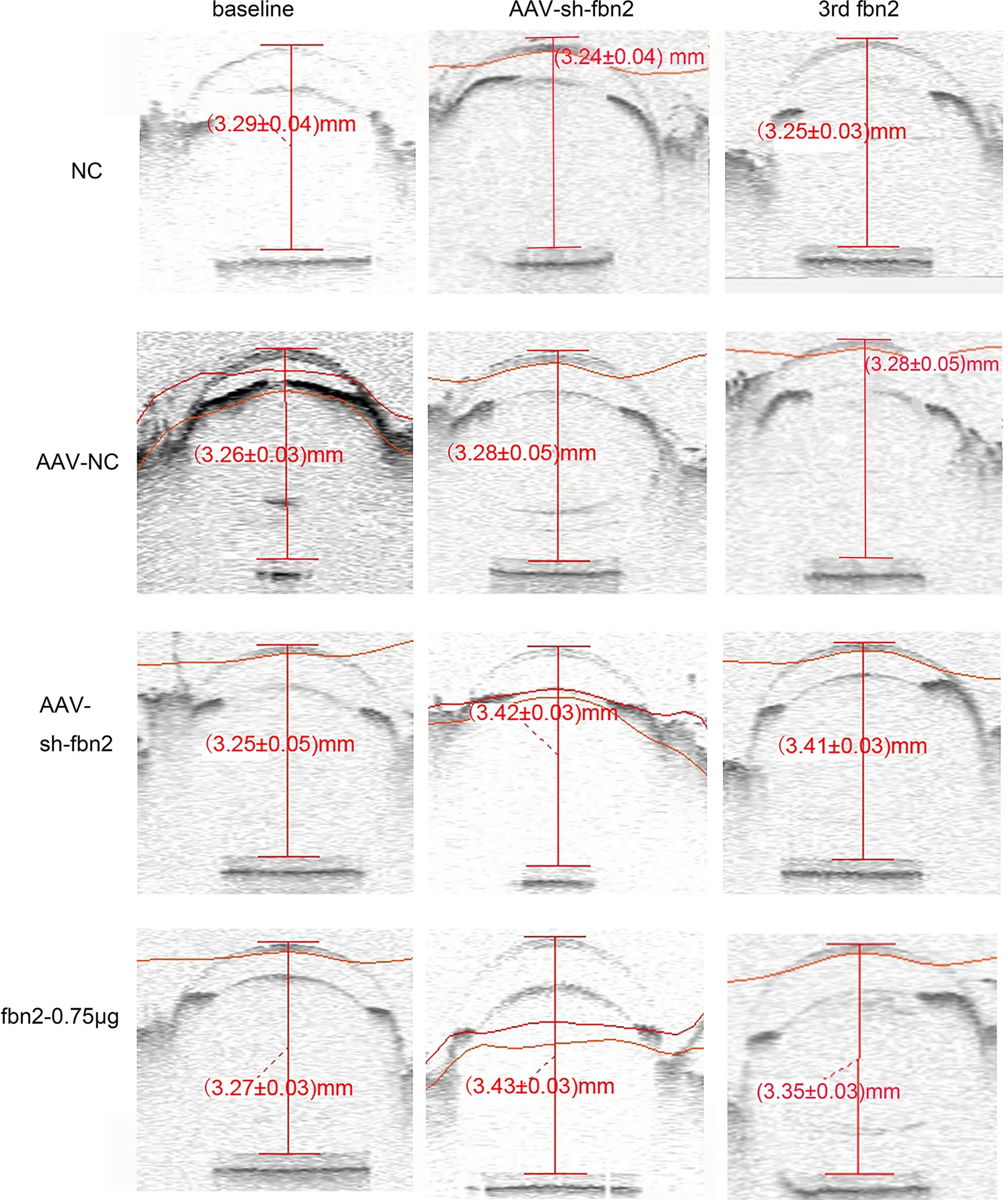
Axial length measured by OCT. NC group: Animals without intervention. AAV-NC group: Animals with an intravitreal injection of AAV empty vector and without any further treatment. AAV-sh-fbn2 group: Animals with an intravitreal injection of AAV-sh-fbn2 and without any further treatment. Fbn2-0.30 μg group: Animals with an intravitreal injection of AAV-sh-fbn2, followed by an intravitreal injection of fbn2 recombinant protein in a dose of 0.30 μg. Fbn2-0.75 μg group: Animals with an intravitreal injection of AAV-sh-fbn2, followed by an intravitreal injection of fbn2 recombinant protein in a dose of 0.75 μg. Fbn2-1.5 μg group: Animals with an intravitreal injection of AAV-sh-fbn2, followed by an intravitreal injection of fbn2 recombinant protein in a dose of 1.50 μg. Fbn2-3.0 μg group: Animals with an intravitreal injection of AAV-sh-fbn2, followed by an intravitreal injection of fbn2 recombinant protein in a dose of 3.00 μg. NC group: Animals without intervention. AAV-NC group: Animals with an intravitreal injection of AAV empty vector and without any further treatment. AAV-sh-fbn2 group: Animals with an intravitreal injection of AAV-sh-fbn2 and without any further treatment. Fbn2-0.30 μg group: Animals with an intravitreal injection of AAV-sh-fbn2, followed by an intravitreal injection of fbn2 recombinant protein in a dose of 0.30 μg. Fbn2-0.75 μg group: Animals with an intravitreal injection of AAV-sh-fbn2, followed by an intravitreal injection of fbn2 recombinant protein in a dose of 0.75 μg. Fbn2-1.5 μg group: Animals with an intravitreal injection of AAV-sh-fbn2, followed by an intravitreal injection of fbn2 recombinant protein in a dose of 1.50 μg. Fbn2-3.0 μg group: Animals with an intravitreal injection of AAV-sh-fbn2, followed by an intravitreal injection of fbn2 recombinant protein in a dose of 3.00 μg.

