# Supplementary material for: Intravitreal injection of fibrillin 2 (Fbn2) recombinant protein for therapy of retinopathy in a retina-specific Fbn2 knock-down mouse model
Source: Sci Rep. 2023 Apr 26;13:6865. doi: 10.1038/s41598-023-33886-6 (PMC10133334; doi:10.1038/s41598-023-33886-6)
Supplement: Supplementary file 1 — Supplementary Information 1. [file 41598_2023_33886_MOESM1_ESM.pdf]

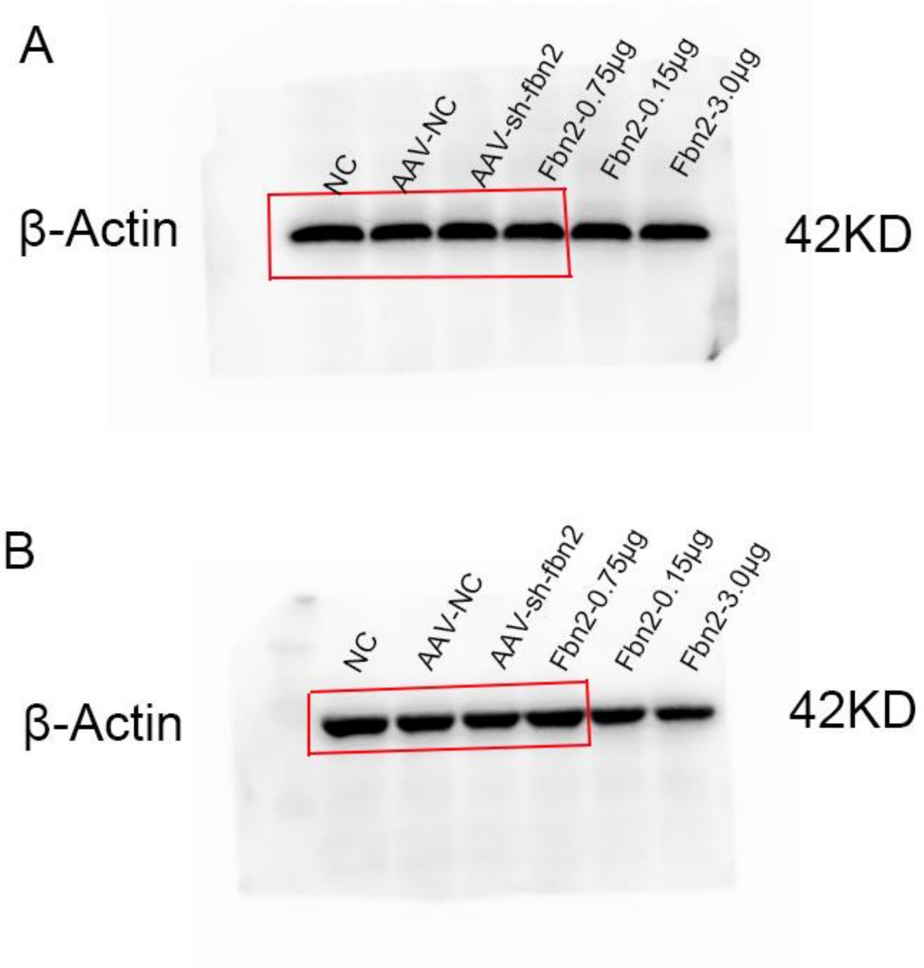

Figure 2. The original blotting of β-Actin. A. Blotting of β-Actin corresponding to fbn2. B. β-Actin blotting corresponding to Tgf-β1 and LTBP-1.

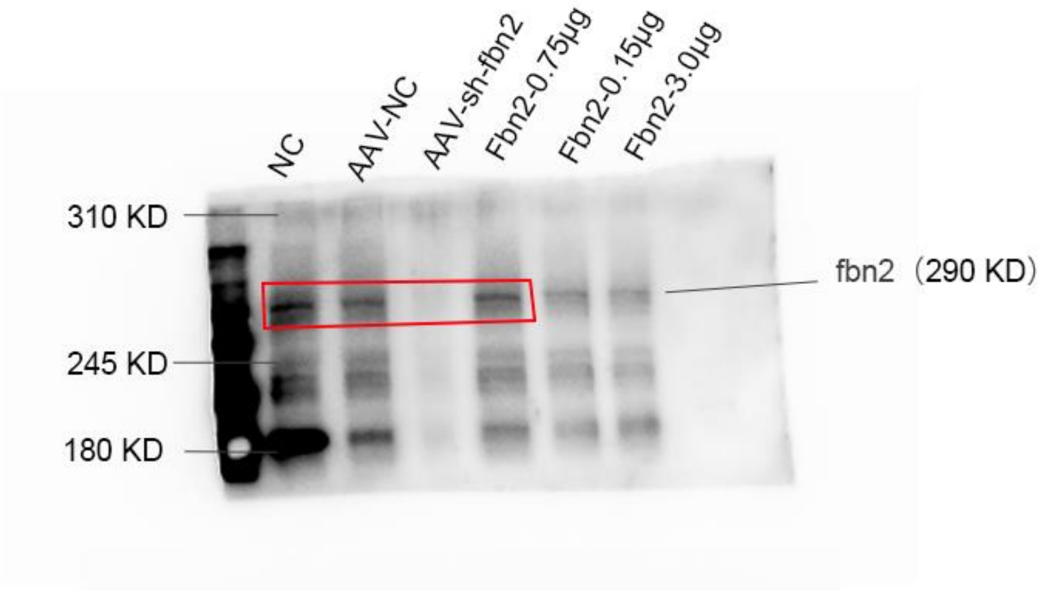

Figure 3. The original blotting of fbn2 (290KD). NC group: Animals without intervention. AAV-NC group: Animals with an intravitreal injection of AAV empty vector and without any further treatment. AAV-sh-fbn2 group: Animals with an intravitreal injection of AAV-sh-fbn2 and without any further treatment. Fbn2-0.30μg group: Animals with an intravitreal injection of AAV-sh-fbn2, followed by an intravitreal injection of fbn2 recombinant protein in a dose of 0.30μg. Fbn2-0.75μg group: Animals with an intravitreal injection of AAV-sh-fbn2, followed by an intravitreal injection of fbn2 recombinant protein in a dose of 0.75μg. Fbn2-0.15μg group: Animals with an intravitreal injection of AAV-sh-fbn2, followed by an intravitreal injection of fbn2 recombinant protein in a dose of 1.50μg. Fbn2-3.0μg group: Animals with an intravitreal injection of AAV-sh-fbn2, followed by an intravitreal injection of fbn2 recombinant protein in a dose of 3.00μg.

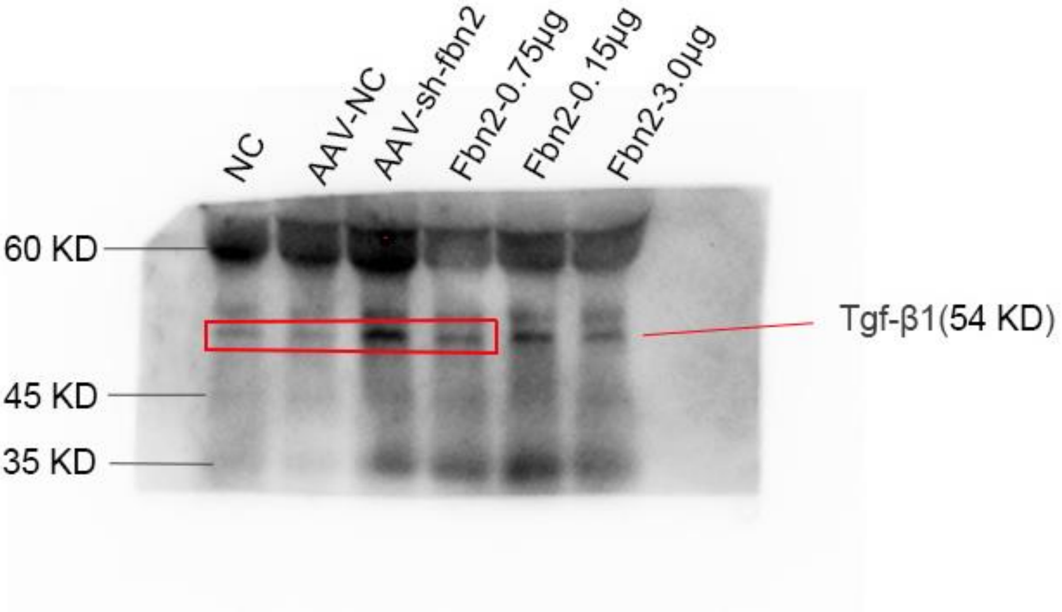

Figure 4. The original blotting of Tgf-β1 (54KD). NC group: Animals without intervention. AAV-NC group: Animals with an intravitreal injection of AAV empty vector and without any further treatment. AAV-sh-fbn2 group: Animals with an intravitreal injection of AAV-sh-fbn2 and without any further treatment. Fbn2-0.30μg group: Animals with an intravitreal injection of AAV-sh-fbn2, followed by an intravitreal injection of fbn2 recombinant protein in a dose of 0.30μg. Fbn2-0.75μg group: Animals with an intravitreal injection of AAV-sh-fbn2, followed by an intravitreal injection of fbn2 recombinant protein in a dose of 0.75μg. Fbn2-0.15μg group: Animals with an intravitreal injection of AAV-sh-fbn2, followed by an intravitreal injection of fbn2 recombinant protein in a dose of 1.50μg. Fbn2-3.0μg group: Animals with an intravitreal injection of AAV-sh-fbn2, followed by an intravitreal injection of fbn2 recombinant protein in a dose of 3.00μg.

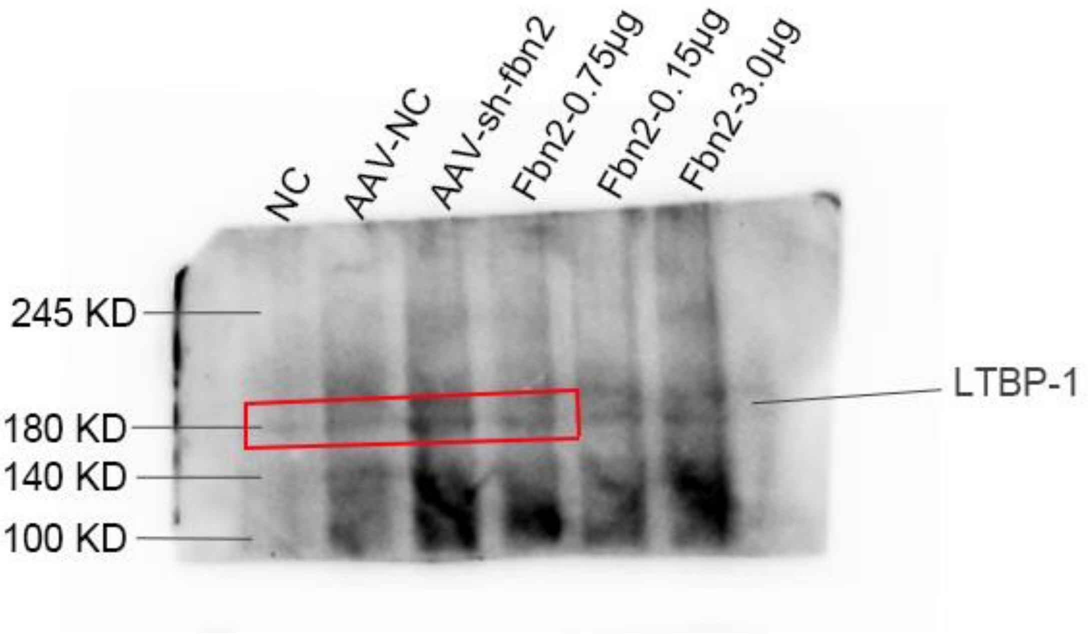

Figure 5. The original blotting of LTBP-1 (180KD). NC group: Animals without intervention. AAV-NC group: Animals with an intravitreal injection of AAV empty vector and without any further treatment. AAV-sh-fbn2 group: Animals with an intravitreal injection of AAV-sh-fbn2 and without any further treatment. Fbn2-0.30μg group: Animals with an intravitreal injection of AAV-sh-fbn2, followed by an intravitreal injection of fbn2 recombinant protein in a dose of 0.30μg. Fbn2-0.75μg group: Animals with an intravitreal injection of AAV-sh-fbn2, followed by an intravitreal injection of fbn2 recombinant protein in a dose of 0.75μg. Fbn2-0.15μg group: Animals with an intravitreal injection of AAV-sh-fbn2, followed by an intravitreal injection of fbn2 recombinant protein in a dose of 1.50μg. Fbn2-3.0μg group: Animals with an intravitreal injection of AAV-sh-fbn2, followed by an intravitreal injection of fbn2 recombinant protein in a dose of 3.00μg.
