# Supplementary material for: Intravitreal injection of fibrillin 2 (Fbn2) recombinant protein for therapy of retinopathy in a retina-specific Fbn2 knock-down mouse model
Source: Sci Rep. 2023 Apr 26;13:6865. doi: 10.1038/s41598-023-33886-6 (PMC10133334; doi:10.1038/s41598-023-33886-6)
Supplement: Supplementary file 2 — Supplementary Information 2. [file 41598_2023_33886_MOESM2_ESM.pdf]

Supple-Table 1 Electroretinogram measurements of pre-experiment in the retina (mean ± standard deviation).

	group	n	Baseline (μV)	AAV-sh-fbn2 (μV)	1st Fbn2 (μV)	2nd Fbn2 (μV)	3rd Fbn2 (μV)	4th Fbn2 (μV)
Rod-b	NC	4	130.60±16.39	140.75±15.58	144.77±14.78	139.99±15.32	140.54±16.44	145.38±28.31
	AAV-NC	4	139.55±19.04	143.61±21.90	137.95±13.89	133.10±12.06	130.22±18.34	134.91±27.73
	AAV-sh-fbn2	4	144.38±15.55	22.27±3.73**	19.53±4.34**	26.32±6.53**	20.32±4.83**	26.25±5.88**
	Fbn2-0.30μg	4	132.87±17.43	18.22±5.64**	22.26±3.77**	30.42±5.88**	31.73±4.79**	31.75±8.79**
	Fbn2-0.75μg	4	140.65±19.58	22. 63±4.99**	28.44±5.67*	69.64±9.64*#	110.32±13.47#	108.33±22.42#
	Fbn2-0.15μg	4	131.88±23.66	17.34±4.73**	24.76±4.77**	40.48±7.33*	67.32±8.54*#	70.00±12.51*#
	Fbn2-3.0μg	4	150.35±16.66	13.11±5.35**	21.54±1.74**	33.69±4.43*	52.32±10.35*#	58.88±15.32*#
Max-a	NC	4	-122.90±12.65	-117.21±14.76	-126.32±13.34	-118.43±18.46	-124.5±13.54	-119.5±17.33
	AAV-NC	4	-110.88±16.66	-120.42±12.85	-118.36±15.32	-123.90±14.44	-119.35±19.34	-111.05±16.71
	AAV-sh-fbn2	4	-112.45±14.53	-14.24±3.86**	-11.74±3.34**	-15.82±3.70**	-11.12±3.34**	-16.17±3.67**
	Fbn2-0.30μg	4	-116.74±13.88	-17.36±4.76**	-14.47±2.34**	-25.04±6.63**	-22.72±8.33**	-28.72±4.33**
	Fbn2-0.75μg	4	-117.7±23.21	-16.33±4.55**	-22.67±4.37**	-70.58±6.88*#	-90.43±16.43#	-84.44±20.42#
	Fbn2-0.15μg	4	-121.54±16.89	-22.32±2.21**	-18.56±3.43**	-30.25±6.33*	-59.27±11.30*#	-65.27±16.68*#
	Fbn2-3.0μg	4	-113.99±14.83	-20.55±7.65**	-20.97±3.47**	-29.77±3.65*	-35.54±5.32*#	-30.90±9.88*#
Cone-b	NC	4	68.44±12.77	75.21±15.44	80.45±5.47	77.44±8.43	73.31±19.03	74.31±12.80
	AAV-NC	4	60.66±9.54	62.90±11.51	77.75±6.39	70.44±5.55	64.03±11.44	70.03±8.55
	AAV-sh-fbn2	4	73.54±10.90	3.89±2.82**	4.09±1.04**	5.53±1.95**	3.97±2.14**	3.48±2.90**
	Fbn2-0.30μg	4	67.54±10.00	4.17±2.57**	6.43±2.85**	7.64±3.53**	6.54±3.44**	8.55±5.44**
	Fbn2-0.75μg	4	61.55±10.69	2.98±3.63**	6.23±2.7	28.18±5.3*#	50.61±17.63#	48.61±19.63#
	Fbn2-0.15μg	4	76.44±18.54	2.87±1.99**	5.45±3.38	15.55±4.03*	28.53±8.07*#	27.57±9.54*#
	Fbn2-3.0μg	4	68.43±14.59	4.66±2.60**	3.99±1.95**	6.06±3.89*	14.66±9.44*#	13.64±8.43*#

Note: * P<0.05 compared with the AAV-NC group; ** P<0.001 compared with the AAV-NC

group; [#] $P<0.05$ compared with the AAV-sh-fbn2 group; [##] $P<0.001$ compared with the AAV-sh-fbn2 group

Rod-b: rod b-wave amplitude (dark adaptation 0.01 ERG). Max-a: the maximal a-wave (dark adaptation 3.0 ERG). Cone-b: cone b-wave amplitude (light adaptation 3.0 ERG).

NC: Animals without intervention. AAV-NC group: Animals with an intravitreal injection of AAV empty vector and without any further treatment. AAV-sh-fbn2: Animals with an intravitreal injection of AAV-sh-fbn2 and without any further treatment. Fbn2-0.30μg: Animals with an intravitreal injection of AAV-sh-fbn2, followed by an intravitreal injection of fbn2 recombinant protein in a dose of 0.30μg. Fbn2-0.75μg: Animals with an intravitreal injection of AAV-sh-fbn2, followed by an intravitreal injection of fbn2 recombinant protein in a dose of 0.75μg. Fbn2-0.15μg: Animals with an intravitreal injection of AAV-sh-fbn2, followed by an intravitreal injection of fbn2 recombinant protein in a dose of 1.50μg. Fbn2-3.0μg: Animals with an intravitreal injection of AAV-sh-fbn2, followed by an intravitreal injection of fbn2 recombinant protein in a dose of 3.00μg.

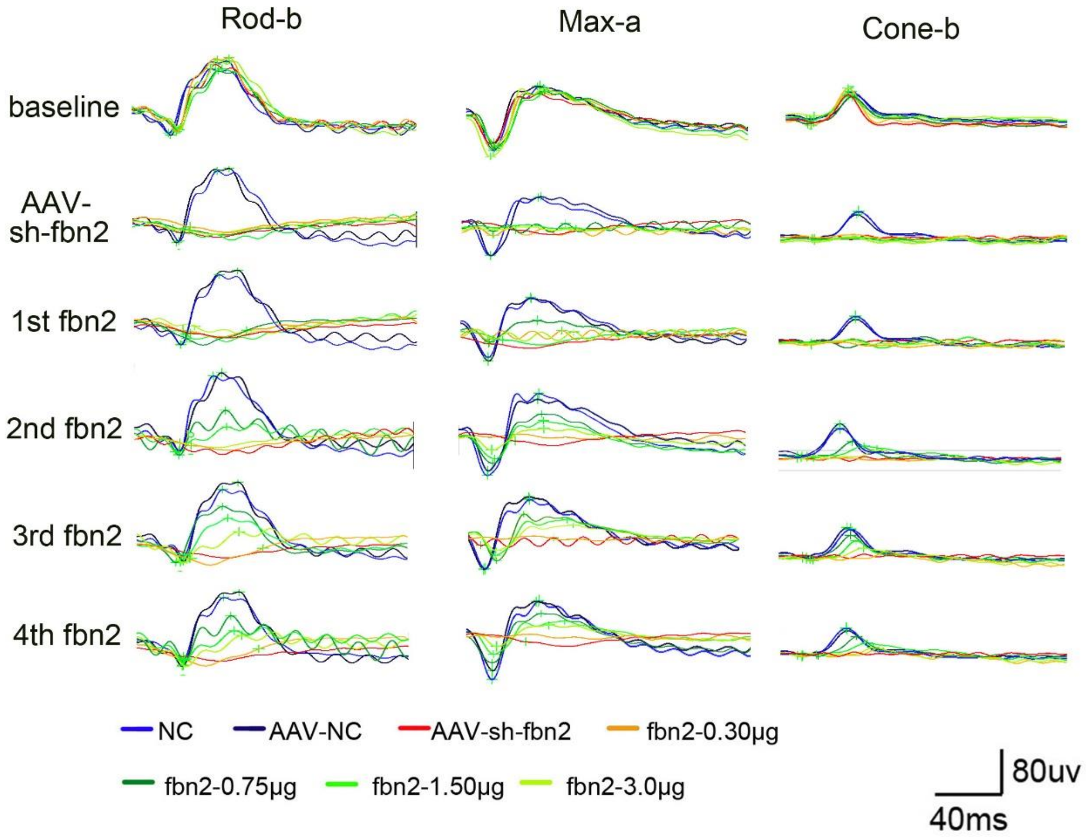

Supple-Figure 1. Electroretinogram measurements of pre-experiment in the retina

NC group: Animals without intervention. AAV-NC group: Animals with an intravitreal injection of AAV empty vector and without any further treatment. AAV-sh-fbn2 group: Animals with an intravitreal injection of AAV-sh-fbn2 and without any further treatment. Fbn2-0.30μg group: Animals with an intravitreal injection of AAV-sh-fbn2, followed by an intravitreal injection of fbn2 recombinant protein in a dose of 0.30μg. Fbn2-0.75μg group: Animals with an intravitreal injection of AAV-sh-fbn2, followed by an intravitreal injection of fbn2 recombinant protein in a dose of 0.75μg. Fbn2-0.15μg group: Animals with an intravitreal injection of AAV-sh-fbn2, followed by an intravitreal injection of fbn2 recombinant protein in a dose of 1.50μg. Fbn2-3.0μg group: Animals with an intravitreal injection of AAV-sh-fbn2, followed by an intravitreal injection of fbn2 recombinant protein in a dose of 3.00μg.
